# Deploying new generation sequencing for the study of flesh color depletion in Atlantic Salmon (*Salmo salar*)

**DOI:** 10.1186/s12864-021-07884-9

**Published:** 2021-07-17

**Authors:** Thu Thi Minh Vo, Tuan Viet Nguyen, Gianluca Amoroso, Tomer Ventura, Abigail Elizur

**Affiliations:** 1 GeneCology Research Centre, University of the Sunshine Coast, Queensland Sunshine Coast, Australia; 2 School of Science, Technology and Engineering, University of the Sunshine Coast, Sunshine Coast, Queensland Australia; 3 Centre for AgriBiosciences, AgriBio, Agriculture Victoria, Victoria 3083 Bundoora, Australia; 4 Petuna Aquaculture, Tasmania 7310 East Devonport, Australia; 5 School of Biotechnology, International University, Viet Nam National University, 700000 Ho Chi Minh City, Vietnam

**Keywords:** RNA-Seq, TruSeq, 3’ mRNA-Seq, Lexogen QuantSeq, Flesh discoloration, Atlantic salmon, Gene expression, Missense mutation

## Abstract

**Background:**

The flesh pigmentation of farmed Atlantic salmon is formed by accumulation of carotenoids derived from commercial diets. In the salmon gastrointestinal system, the hindgut is considered critical in the processes of carotenoids uptake and metabolism. In Tasmania, flesh color depletion can noticeably affect farmed Atlantic salmon at different levels of severity following extremely hot summers. In this study, RNA sequencing (RNA-Seq) was performed to investigate the reduction in flesh pigmentation. Library preparation is a key step that significantly impacts the effectiveness of RNA sequencing (RNA-Seq) experiments. Besides the commonly used whole transcript RNA-Seq method, the 3’ mRNA-Seq method is being applied widely, owing to its reduced cost, enabling more repeats to be sequenced at the expense of lower resolution. Therefore, the output of the Illumina TruSeq kit (whole transcript RNA-Seq) and the Lexogen QuantSeq kit (3’ mRNA-Seq) was analyzed to identify genes in the Atlantic salmon hindgut that are differentially expressed (DEGs) between two flesh color phenotypes.

**Results:**

In both methods, DEGs between the two color phenotypes were associated with metal ion transport, oxidation-reduction processes, and immune responses. We also found DEGs related to lipid metabolism in the QuantSeq method. In the TruSeq method, a missense mutation was detected in DEGs in different flesh color traits. The number of DEGs found in the TruSeq libraries was much higher than the QuantSeq; however, the trend of DEGs in both library methods was similar and validated by qPCR.

**Conclusions:**

Flesh coloration in Atlantic salmon is related to lipid metabolism in which apolipoproteins, serum albumin and fatty acid-binding protein genes are hypothesized to be linked to the absorption, transport and deposition of carotenoids. Our findings suggest that Grp could inhibit the feeding behavior of low color-banded fish, resulting in the dietary carotenoid shortage. Several SNPs in genes involving in carotenoid-binding cholesterol and oxidative stress were detected in both flesh color phenotypes. Regarding the choice of the library preparation method, the selection criteria depend on the research design and purpose. The 3’ mRNA-Seq method is ideal for targeted identification of highly expressed genes, while the whole RNA-Seq method is recommended for identification of unknown genes, enabling the identification of splice variants and trait-associated SNPs, as we have found for *duox2* and *duoxa1*.

**Supplementary Information:**

The online version contains supplementary material available at 10.1186/s12864-021-07884-9.

## Background

The unique pink-red flesh color of salmon is considered to be a decisive factor for its marketabilty as it tends to be associated with freshness and quality. This makes flesh color a key criterion in the salmon farming industry [1]. In the wild the muscle pigmentation of some salmonids is the result of the accumulation of carotenoids, primarily astaxanthin (Ax), derived from the consumption of algae which are Ax main producers, and/or crustaceans which fed on the algae [2]. The muscle pigmentation of farmed salmon relies on a synthesized form of carotenoids included in commercial diets [2]. Due to its hydrophobicity, Ax is not solubilized in the aqueous environment of the fish gastrointestinal system [3]. Several studies suggest that the addition of dietary lipids tends to enhance the deposition of Ax in salmon flesh [4, 5]. Changing the dietary fatty acid composition could influence the solubility of Ax in the lipid phase [3, 6].

Several studies examined the effect of temperature on the growth and development of Atlantic salmon as well as other salmonids [7–9]. The optimal temperature range for Atlantic salmon lies between 12 and 18 °C [7]. Atlantic salmon display behaviors that are adaptive to the daily fluctuations in environmental temperature and dissolved oxygen in farming sea cages. Individual fish tend to adjust their depth to stay at the optimal temperature of 12˚C to 18˚C and show a trend of avoiding low dissolved oxygen [10, 11]. Other studies have shown that exposure to temperatures above the upper optimal temperature significantly affected growth performance by reducing feed intake [9, 12, 13]. Therefore, the elevated summer temperatures experienced by Atlantic salmon grown in Tasmania may affect the fish physiology by inducing a stress response [9, 14]. This is evident at the molecular level by the presence of altered heat shock protein and antioxidant pathways found in salmon exposed to high water temperatures [15–18]. In recent years, global warming has been marked as a key factor with potential devastating impacts on the aquaculture industry, especially in cold-blooded fish [19]. This led to an increased focus on investigating the effect of elevated temperature on the flesh color of salmonids such as Atlantic salmon [18]. Recently, Grunenwald, Adams [20] were able to induce a depletion of color in Atlantic salmon muscle as a result of short term thermal stress. In Tasmania, farmed Atlantic salmon can be exposed to water temperatures of 20˚C and above for extended periods during summer, which can exacerbate flesh color depletion [21]. In 2015-16 Tasmania reported an unprecedented extreme hot summer, during which farmed salmon experienced water temperatures of over 20^o^C, which negatively impacted on both performance and flesh colour (i.e. in some cases flesh colour was almost completely depleted) [20, 21].

Over a decade ago, next-generation sequencing (NGS) has been applied in transcriptomic analysis [22, 23] and has since evolved into a universally-applied tool in molecular biology. Using NGS technologies, RNA-sequencing (RNA-Seq), can be aligned to the reference genome or *de novo* assembled, making it a widely used tool also for non-model organisms [24, 25]. Not only does it enable characterization of the transcriptome’s complexity, but RNA-Seq also enables quantification of gene expression level, calling alternative splicing variations and single nucleotide polymorphisms (SNPs) [24, 26]. In recent years, the cost of NGS analysis decreased considerably, further contributing to its popularity [27]. To date, Illumina has been the most widely used sequencing platform. Using a fluorescence-based reading of multiple short nucleotide sequences, it enables unparalleled output, both in terms of sequencing depth and quality [28, 29]. Although the sequencing unit price is steadily decreasing [24], the expense of library preparation is still considerable [27], and the Illumina protocol for sample library construction (TruSeq, which is considered as a gold standard RNA-seq protocol) is time and labor-intensive [24]. These factors limit the number of samples sequenced using this approach in any given study.

Several library preparation methods have been developed for Illumina RNA-seq, bringing about various choices for users [30]. Lexogen’s QuantSeq 3’ mRNA library preparation (QuantSeq) benefits include reduced price, time and labor in comparison with the standard RNA-Seq [31]. QuantSeq is a quick and straightforward protocol that creates a library of sequences close to the 3’ end of polyadenylated RNAs from only 0.5–500 ng of total RNA input [31]. In addition, there is no need to fragment the mRNA prior to cDNA synthesis [32]. Due to producing only one fragment per transcript, the number of reads aligned to a given gene is the percentage of its expression, regardless of the transcript length [31]. Hence, QuantSeq requires lower sequencing depth than the standard TruSeq and allows for pooling many more libraries on one lane of the flow cell for multiplex sequencing [31–33]. The shortfall of Quantseq is that it does not enable calling alternative splicing variations and SNPs.

In this study, we compared Illumina’s TruSeq kit, the standard RNA-Seq (refered to as TruSeq) and Lexogen’s QuantSeq kit, the 3’ mRNA-Seq method (QuantSeq) for an Atlantic salmon flesh color investigation. Firstly, QuantSeq was used to compare differentially expressed genes (DEGs) between four different flesh color phenotypes. To confirm the results and to analyze whether there are any differences between TruSeq and QuantSeq, TruSeq was used on two chosen sample subsets from two flesh color phenotypes. The sequencing results from TruSeq and QuantSeq libraries were compared to understand their relative benefits and drawbacks.

## Results

### Library preparation and RNA-sequencing

Post reads quality control, an average of 1.9 million and 21.8 million clean reads were obtained per sample for the QuantSeq and TruSeq libraries, respectively. There was not a marked difference in read mapping rate between the sequences from the two sources, with 85 and 88 % of the reads from QuantSeq and TruSeq, respectively, aligning to the salmon genome (data not shown).

### Gene body coverage

**Fig. 1:**
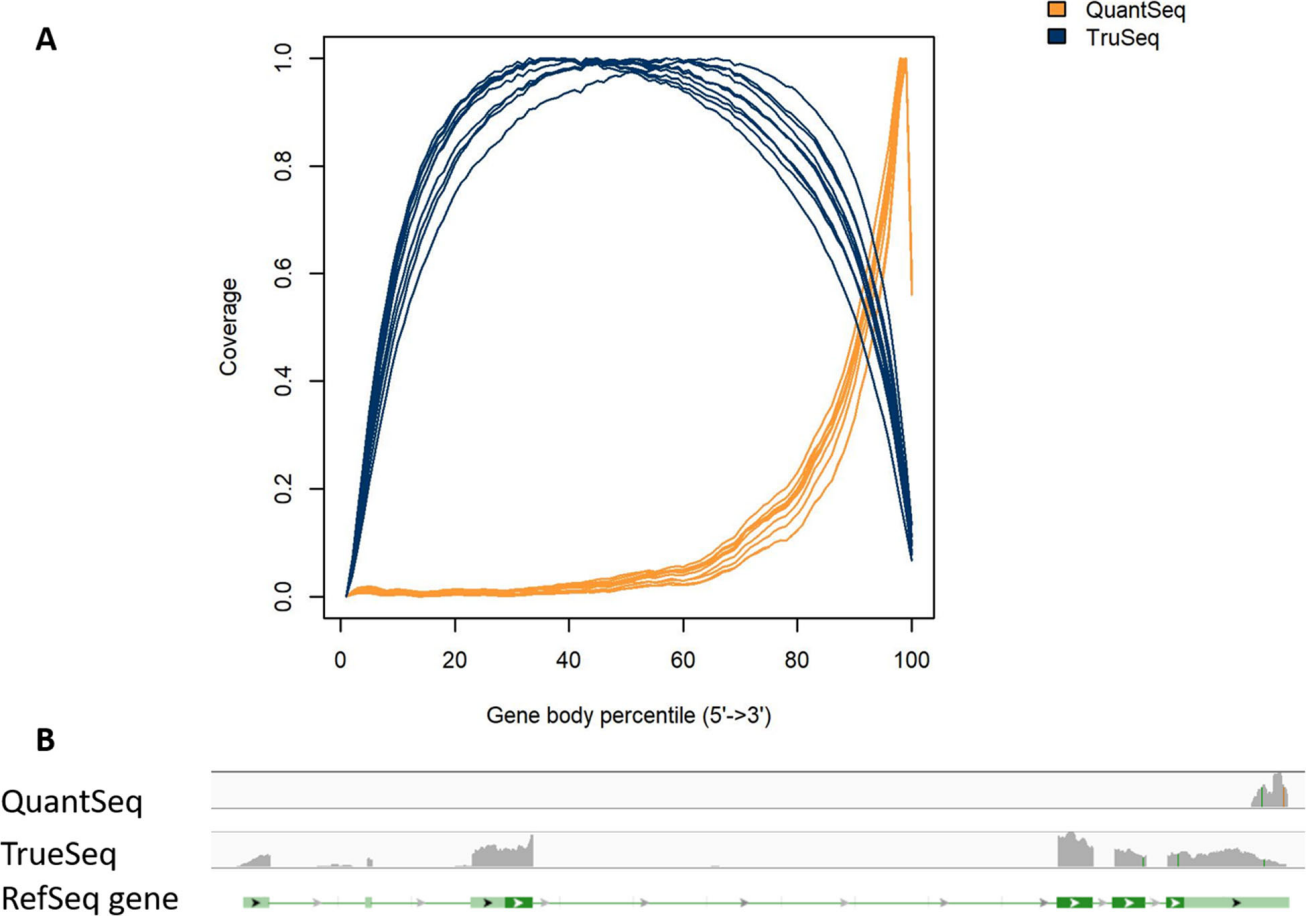
Gene body coverage (**A**) and *SH2 domain-containing protein 4A-like* (***sh24a***) gene body coverage (**B**) from the QuantSeq and TruSeq libraries. Each line in. **A** represents the coverage of mapped reads from every library along with transcripts from 5’ to 3’ ends in the whole Atlantic salmon genome. **B** shows the distribution of mapped reads from QuantSeq and TruSeq libraries on the exons of Sh2a4 gene from 5’ to 3’ end

The distribution of mapped reads along the transcripts is presented in Fig. 1A. As expected, QuantSeq-Lexogen reads map mainly to the 3’ end, while TruSeq-Illumina reads covered the transcripts entirely, with a slight decrease at the 5’ and 3’ ends.

To illustrate the gene body coverage at the single gene level, we used *SH2 domain-containing protein 4A-like* (*sh24a*), where the gene body coverage showed a clear difference in mapping positions between two libraries (Fig. 1B). The QuantSeq reads covered only the last exon at 3’ end, while TruSeq reads covered all exons of the gene with only a slight decline in the exon at the 5’ end. Therefore, the QuantSeq method could not detect gene isoforms correctly in multi-exonic genes with long transcripts and alternative splicing.

### Distribution of reads on different transcript length

**Fig. 2:**
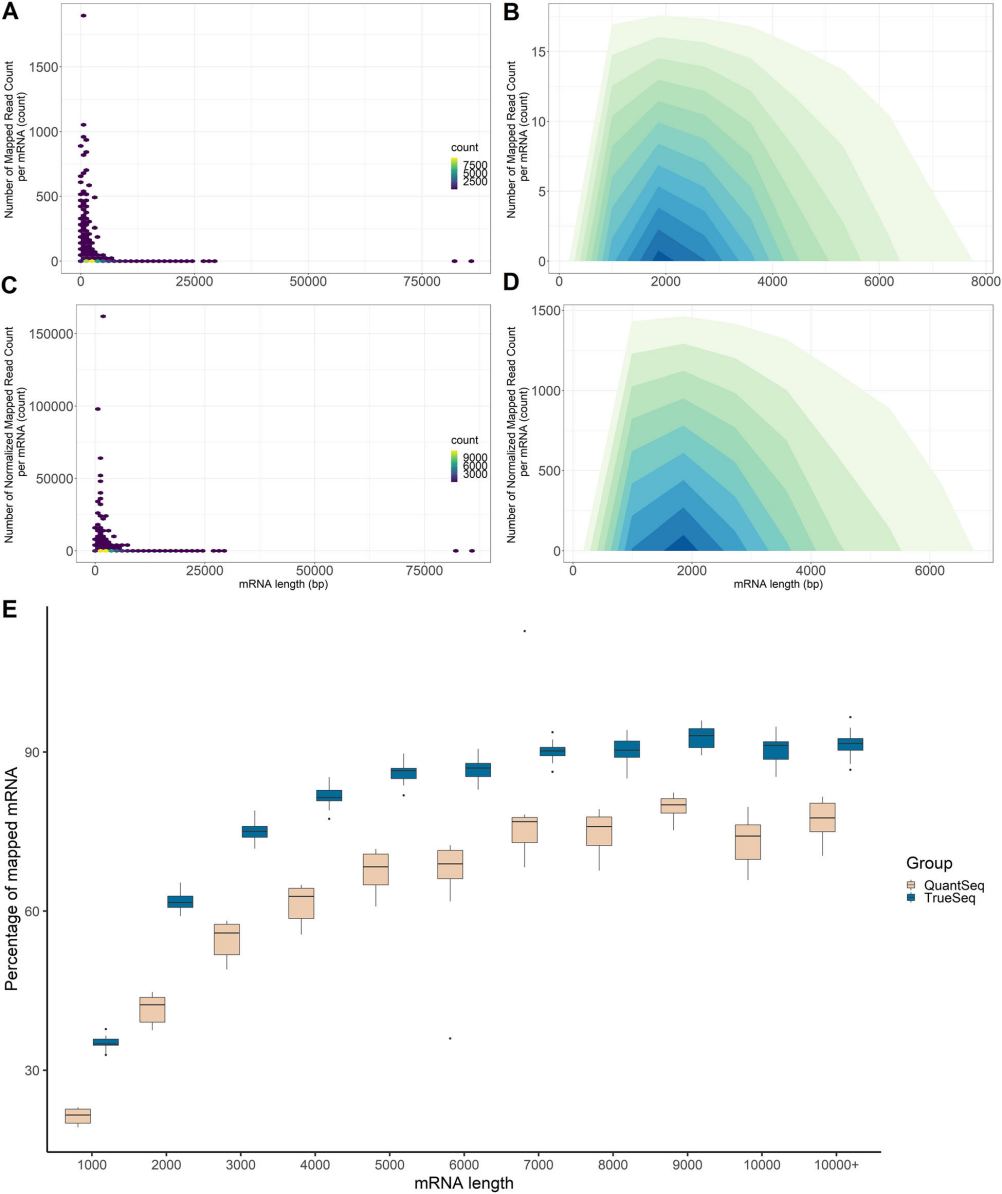
Distribution of mapped mRNA length and percent of mapped mRNA in different size classes. The count of reads for every single mRNA of different mRNA length in QuantSeq (**A**) and TruSeq (**C**); Density of mapped reads per gene of different mRNA length in QuantSeq (**B**) and TruSeq (**D**), darker color indicates higher scatter density; percentage of mapped mRNA of different lengths in QuantSeq and TruSeq libraries (**E**)

Overall, the proportion of mapped genes was similar between QuantSeq and TruSeq libraries (Fig. 2 A and C). Most reads mapped to mRNA smaller than 8000 bp. Figure 2 A and C show that for each gene, TruSeq showed 100 fold higher number of mapped reads (following normalization), consistent with this technology offering a much higher depth of sequencing. In both libraries, most reads mapped to mature mRNA around the size range of 2,000 bp length with Quantseq showing less than 17.5 reads/gene (Fig. 2B) compared with TruSeq with about 1500 normalized mapped read count/gene (Fig. 2D). For the shorter mature mRNAs (< 2,000 bp) a higher read count was detected with the Quantseq libraries (up to 1,000 read count) (Fig. 2 A) and up to 100,000 read count for the TruSeq libraries. Figure 2E shows the percent of genes per size class that were detected with each library. It illustrates that the depth of sequencing affects the percent of genes identified and as can be expected, there were more expressed genes detected when the sequencing depth is much higher.

### Differentially expressed genes

**Fig. 3:**
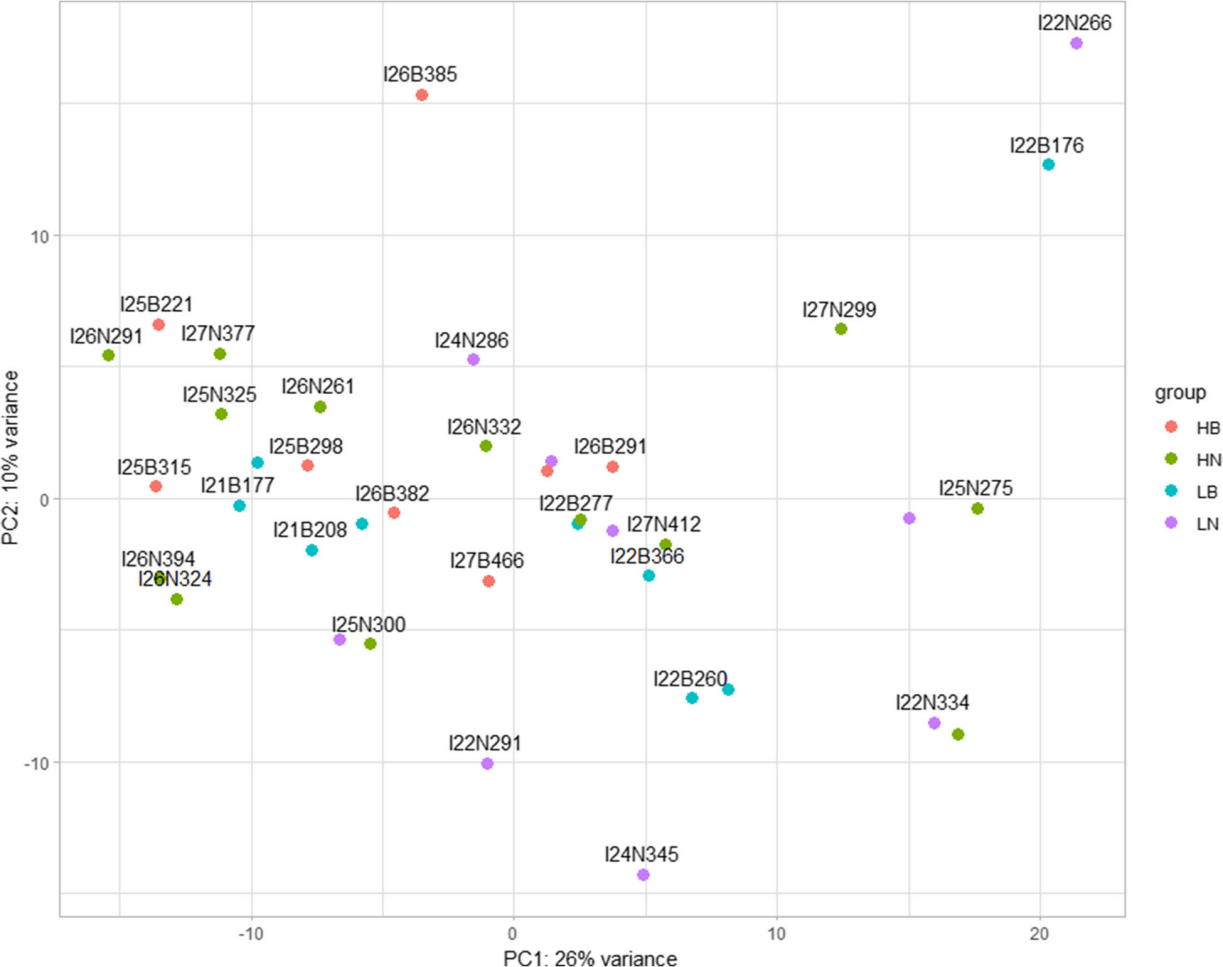
Principal component analysis (PCA) plot visualizing the variation between the expression in 39 samples from 4 different phenotype groups. HN: high-no banding; HB: high-banded; LB: low-banded; LN: low-no banding

Four comparisons between the 39 Quantseq libraries (HN vs. HB, HN vs. LN, HB vs. LB, and LN vs. LB) showed very high variability in expression levels between individuals, and there were only three DEGs upregulated in HB fish as compared to LB fish. These include CAP-Gly domain-containing linker protein 1-like, bleomycin hydrolase and SH3 domain binding glutamate-rich protein like. None of the flesh color phenotype present any clear clustering in the PCA plot (Fig. 3). This result shows that DEGs relating to flesh color phenotype does not manifest in low depth of sequencing despite having many replicates per treatment (from 8 to 13 replicates). To examine the question if the lack of differential expression between the two phenotypes is a result of the shallow sequencing depth, the sequencing was repeated using Illumina TruSeq Library Preparation which provides higher sequencing depth.

**Fig. 4:**
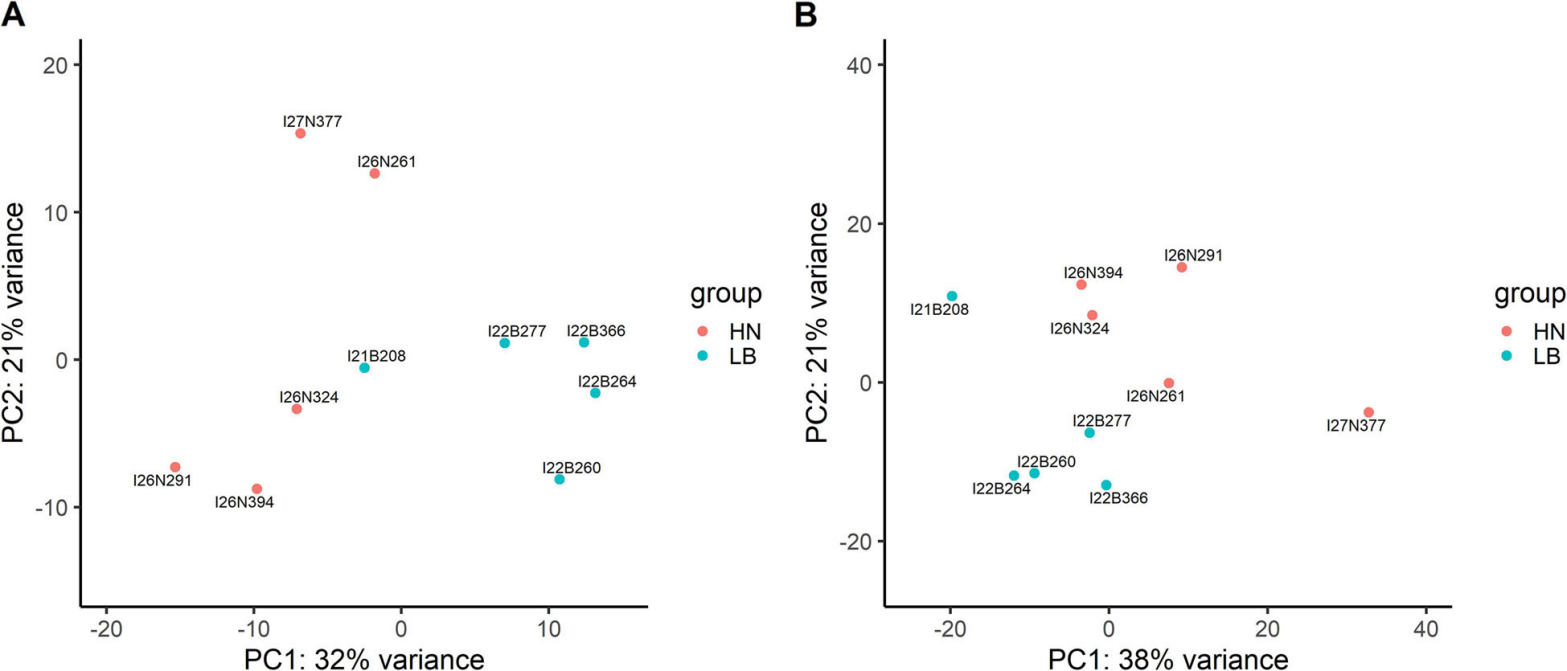
Variation between ten samples from two flesh color phenotypes visualized in PCA plot. **A**: QuantSeq library; **B**: TruSeq library

**Fig. 5:**
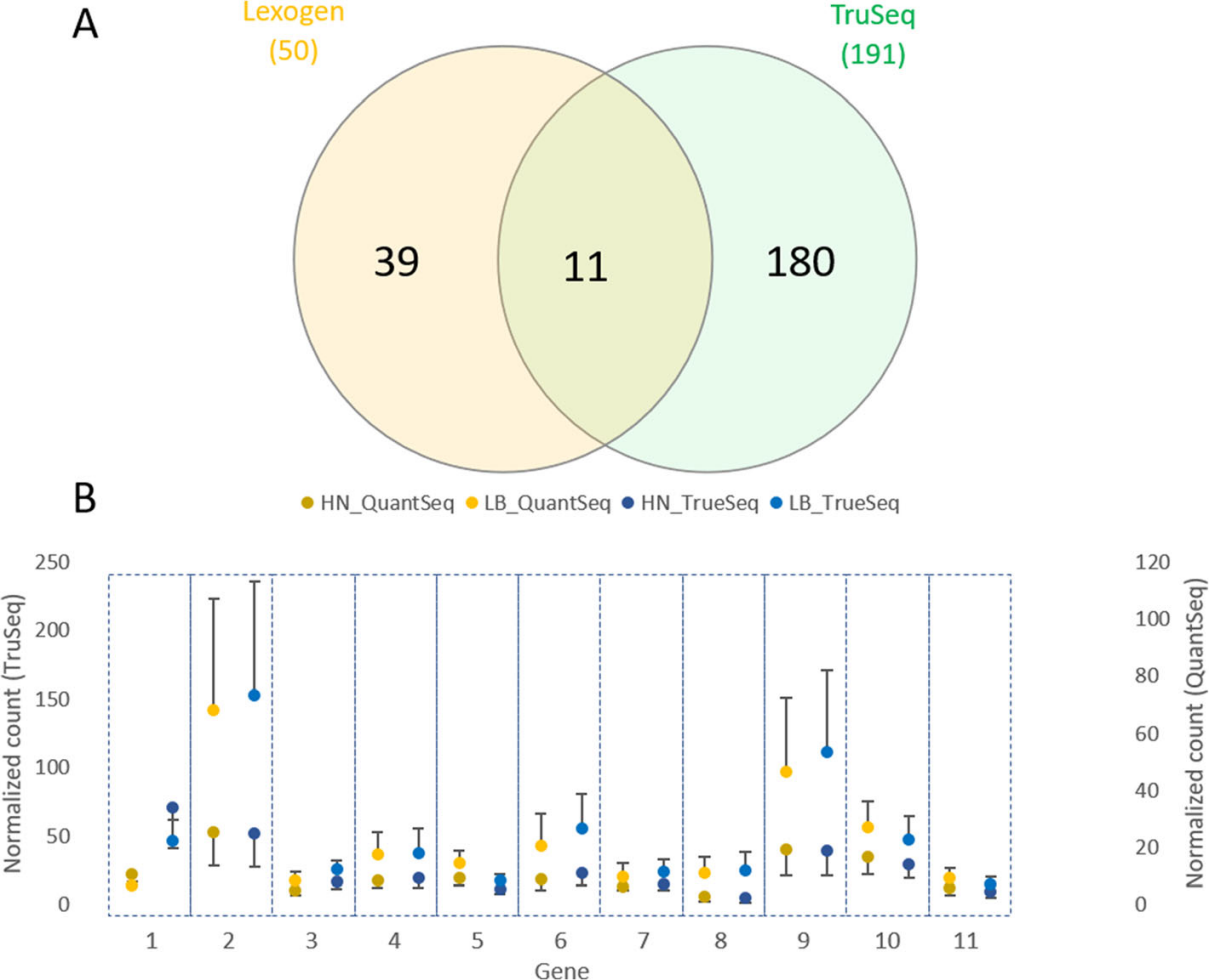
The differential gene expression in the two libraries: (**A**) Venn diagram of DEGs from TruSeq and QuantSeq libraries (**B**) the variation of expression within biological replicates of the 11 DEGs shared in the QuantSeq and TruSeq data. HN- high color no banding, LB- low color, banded. HN_QuantSeq: the normalised counting average of HN subset samples in each DEGs; LB_QuantSeq: the normalised counting average of LB subset samples in each DEGs; HN_TruSeq: the normalised counting average of HN subset samples in each DEGs; LB_TruSeq: the normalised counting average of HN subset samples in each DEGs; The number of DEGs is based on the order in Table 1

Using the same ten samples in two group HN and LB, a comparison of the effectiveness of QuantSeq and TruSeq libraries was conducted. When the PCA plots of sequencing results for the two subsets of samples were compared, there were two significant clusters based on flesh color phenotypes in both QuantSeq and TruSeq (Fig. 4 A & B). In the TruSeq data, 191 DEGs were identified while only 50 DEGs were identified from the QuantSeq data when the two subsample groups were compared. Out of these, only 11 DEGs overlapped between the two methods and all had high normalized counts (Fig. 5). These genes relate to oxidative stress and/or immune response (Table 1). Interestingly, the high variation in expression of these DEGs showed a similar pattern in the TruSeq and QuantSeq datasets. The fact that these 11 DEGs showed the same pattern with the two library methods used instil more confidence in the validity of these DEGs. Forty-eight (96 %) and 165 (86.4 %) DEGs (QuantSeq and TruSeq, respectively), had GO annotation (Supplementary 1). Due to higher number of DEGs, there were more GO terms annotated in TruSeq library than QuantSeq.

**Table. 1:** Eleven differentially expressed genes that overlapped between the TruSeq and QuantSeq libraries

No.	Function	Category
1	*heme oxygenase-like*	stress response (heavy metal and hypoxic stress)
2	*ladderlectin-like*	immune response
3	*cytochrome b-245 light chain-like*	immune response, stress response (ROS, hypoxia)
4	*unconventional myosin-Ib-like*	structural protein, cell proliferation
5	*arachidonate 5-lipoxygenase-activating protein*	immune response
6	*ES1 protein homolog, mitochondrial-like*	metabolic process
7	*sulfide:quinone oxidoreductase, mitochondrial-like*	sulfur metabolism
8	*dual oxidase 2-like*	stress response
9	*CD209 antigen-like*	immune response
10	*GDP-L-fucose synthase*	metabolic process
11	*carcinoembryonic antigen-related cell adhesion molecule 6-like*	immune response

**Fig. 6:**
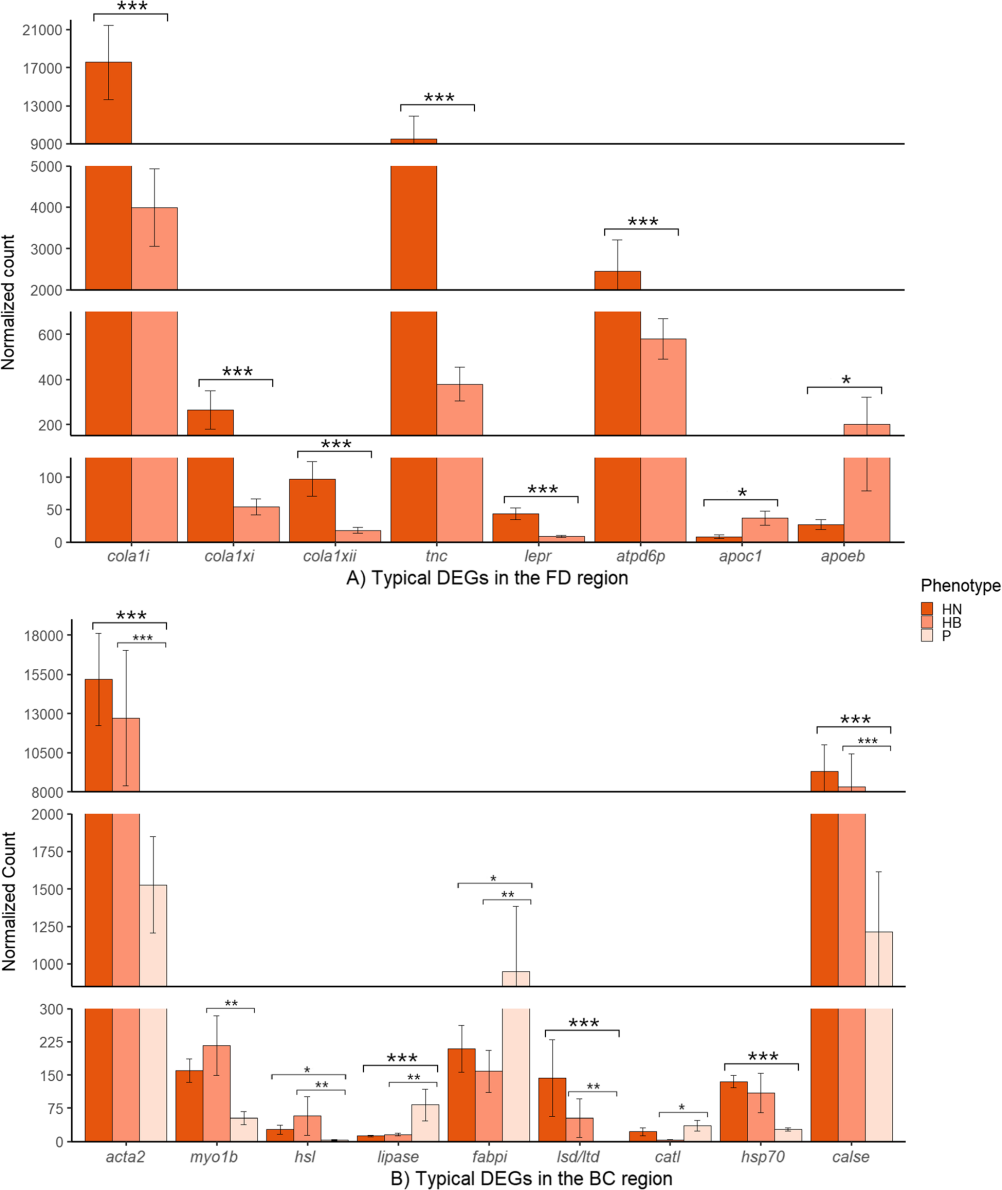
Analysis comparing HN and LB phenotypes. Subset of DEGs of interest identified in the transcriptiomes from the two library preprations. (**A**) Expression pattern of eight genes identified in the QuantSeq library: *ferritin-m (fertn-m)*, involved in iron ion transport; *cytochrome c oxidative subunit 6 A mitochondrial-like (cypc6a)*, involved in oxidation-reduction process; *cathepsin L1 (catl1)* and *cathepsin B (catb)* involved in apoptosis and muscle degradatio*n*; *apolipoprotein A1 (apoa1), apolipoprotein C1 (apoc1), serum albumin 2 (alb2), fatty acid-binding protein 1 (fabp1)* involved in lipid metabolism; (**B**) Expression pattern of seven genes identified in the TruSeq library: cyp450 gene family *cyp2k1, cyp3a27 and cyp1b1*, involved in oxidation-reduction process; *gastrin-releasing peptide (grp)* involved in regulation of feeding, and *tmprss9*, *dual oxidase 2-like (duox2), dual oxidase maturation factor 1-like (duoxa1)* involved in SNP analysis. Asterisks (* and **) indicate significant difference between the HN and LB phenotypes at* P < 0.05 and P < 0.01, *respectively

In the QuantSeq library, we detected 50 DEGs (Supplementary 2a) related to metal ion transport, oxidation-reduction process, immune responses and lipid metabolism. Regarding iron ion transport, *ferritin middle subunit (fertn-m)* was upregulated in HN fish. Also, *cytochrome (CY) c oxidative subunit 6 A mitochondrial-like (cypc6a)* in LB fish and *cathepsin L1-like (catl1)* and *cathepsin B (catb)* were upregulated in HN fish. With regards to lipid metabolism, several genes related to lipid binding and transporters are upregulated in HN fish including *apolipoprotein A-I (apoa1), apolipoprotein C-I (apoc1), serum albumin 2 (alb2)* and *fatty acid-binding protein 1, liver (fabp1)* (Fig. 6 A).

DEGs detected in the TruSeq library (Supplementary 2b) that were different to those found in the Quantseq library, were also linked with metal ion transport, oxidation-reduction process and immune responses. Regarding the oxidation-reduction process, *cypc6a* was not a DEG, but three other genes belonging to the CYP450 family were upregulated in HN fish. These include *CYP450 2K1-like (cyp2k1), CYP450 3A27-like (cyp3a27) and CYP450 1B1-like (Cyp1b1)* (Fig. 6B). In our present study, *gastrin-releasing peptide (grp)* was upregulated in LB fish, which did not consume food well, while Grp expression in HN fish was lower despite the fact they grew and displayed food uptake.

### RT-qPCR validation

**Fig. 7:**
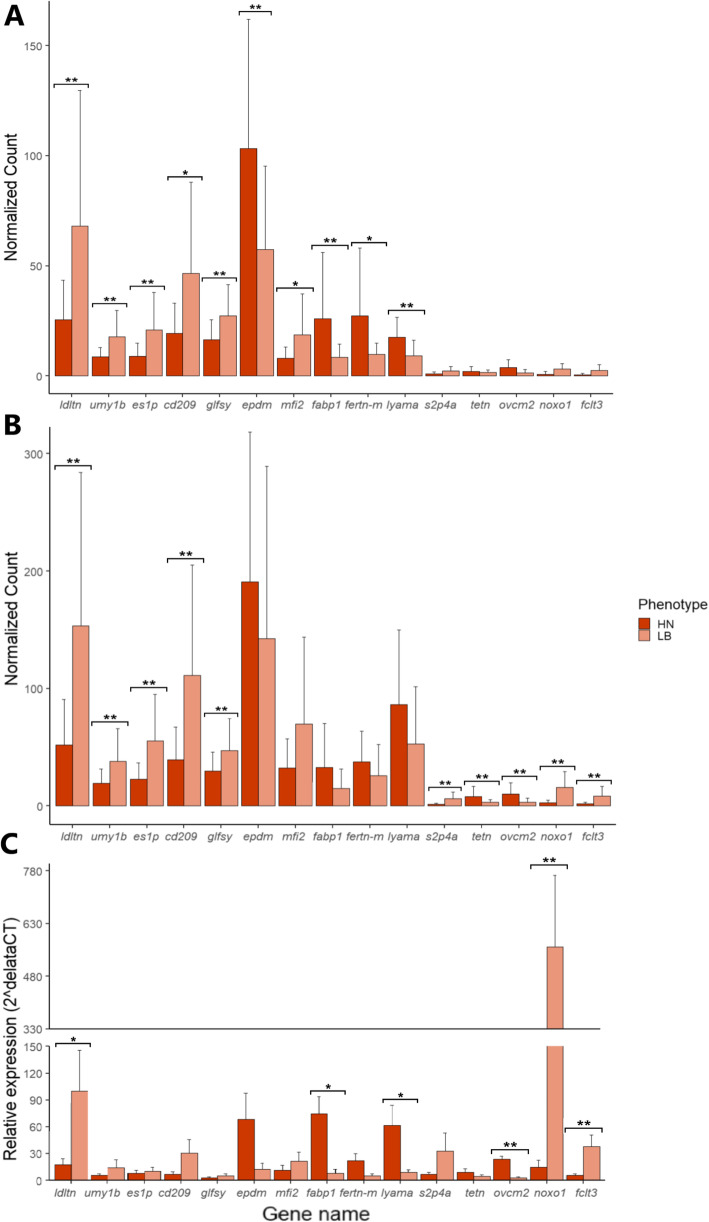
Comparison of variations within biological replicates of 15 DEGs from the Quantseq and Trueseq libraries and RT-qPCR validation using the same biological samples; Gene expression in (**A**) QuantSeq library; (**B**) TruSeq library; (**C**) RT-qPCR. From left to right, the first five genes were significantly different between HN and LB in both library methods but only *ldtn* shows a significant difference in RT-qPCR assay; the next five genes showed significant differences only in the QuantSeq library analysis, and only two of those (*fabp1* and *lyama*) were significantly different in RT-qPCR; the last five genes showed significant differences only in the TruSeq library, and only three out of these five genes (*ovcm2, noxo1* and *fclt3*) were significantly different using the RT-qPCR assay. Asterisks (* and **) indicate significant difference between the HN and LB phenotypes at P < 0.05 and P < 0.01, respectively

Further validation using qPCR was performed on 15 DEGs; five that were DEGs only in the QuantSeq data, 5 that were DEGs only in the TruSeq data and 5 that were DEGs in both datasets. The expression pattern of fifteen genes was validated using RT-qPCR, in comparison with their expression pattern calculated using the QuantSeq and TruSeq libraries (Fig. 7). While the trend of expression was very similar between DEGs measured in both libraries and the qPCR validation, there were only 6 out of 15 of genes that were significantly differentially expressed when assessed using qPCR. Out of five DEGs that belonged to the group significantly expressed in both libraries, there is only one gene, *ldtn*, which is significantly differentially expressed according to the RT-qPCR result (*P < 0.05*). From the five DEGs detected in the QuantSeq data, two genes, *fabp1* and *lyama*, were validated as DEGs using RT-qPCR, and three out of five TruSeq data DEGs were validated as DEGs by RT-qPCR (*P < 0.05*).

### SNP analysis

Using Illumina data with high sequencing depth, we found several SNP variants specific to samples of high color-no banding (HN) or low color-banded (LB) traits. In this analysis, we focused on variants located on DEGs or carotenoid-related genes and may cause a missense mutation, assuming that replacement of amino acids could affect protein structure or assembly rate. Among 191 DEGs identified in the Truseq library, there were 5 DEGs with nine missense mutations and 2 DEGs with three missense mutations found in HN and LB fish, respectively. In HN fish, nine missense mutations were detected in *fucolectin-3-like*, *serine-aspartate repeat-containing protein F-like, transmembrane protease serine 9-like, dual oxidase 2-like*, and *dual oxidase maturation factor 1-like*. Three missense mutations were identified in LB fish in *fc receptor-like protein 5* and extended *synaptotagmin-like protein 2* in LB fish.

Most carotenoid-related genes did not have any variants that can be considered reliable SNPs because the read depth on these positions was low (less than ten reads), or the alternative allele count was much lower than the reference allele count (20 % or less). Reliable SNPs were considered if they were found in at least three out of five individuals of only one of the two assessed phenotypes. In 13 carotenoid-related genes in the HN fish, missense mutations were identified. We found Pro21Thr missense mutation, located on *ATP-binding cassette, sub-family G (white), member 8 (abcg8)* that occurred in three of five heterozygous individuals of high color-no banding group.

**Fig. 8:**
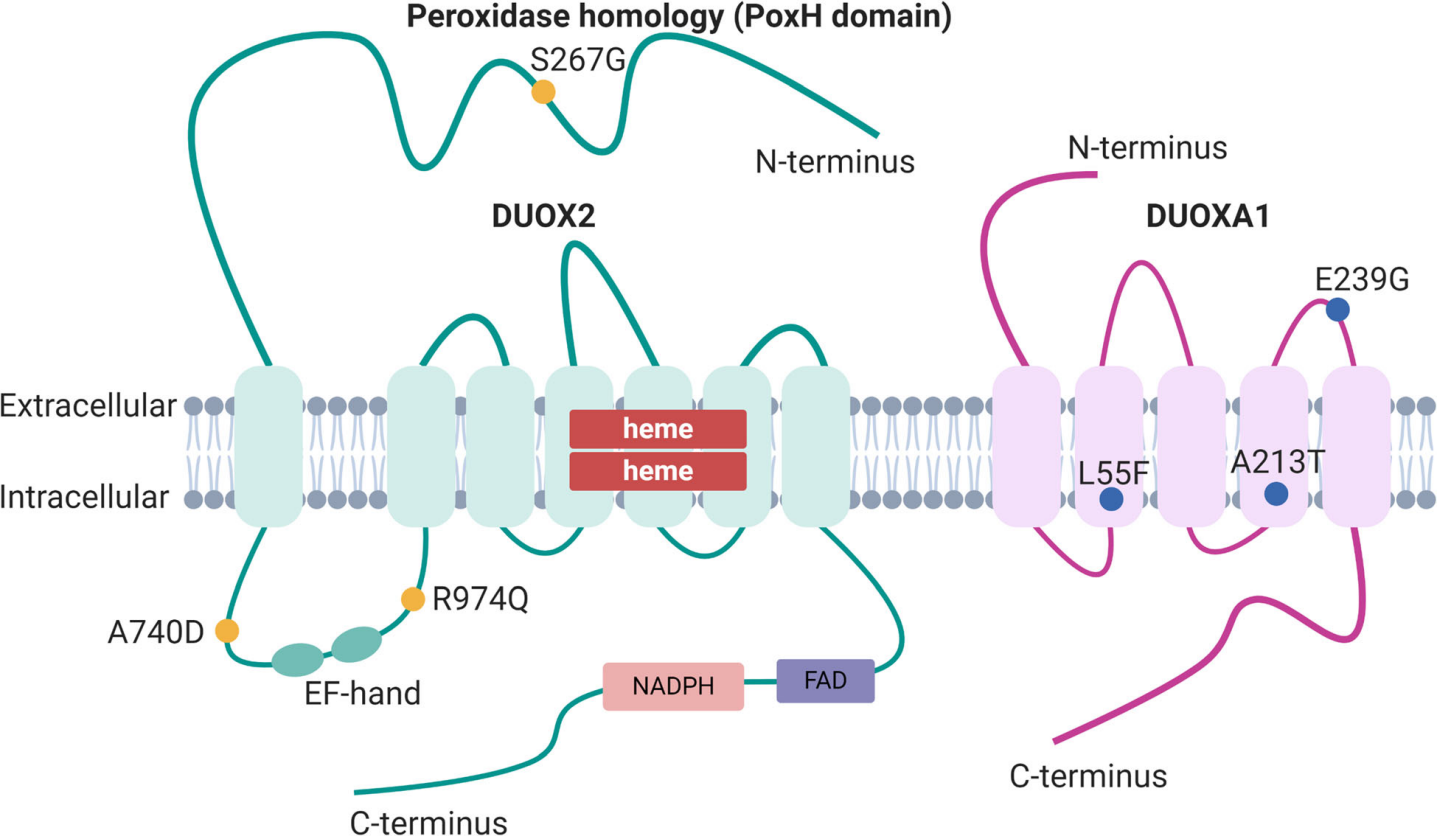
Schematic illustration of SNPs on ***dual oxidase 2*** and ***dual oxidase maturation factor 1***. *duox2: dual oxidase 2; duoxa1: dual oxidase maturation factor 1*. S267G, A740D, R974Q: missense mutations in DUOX2; L55F, A213T, E239G: missense mutations in DUOXA1. Created with BioRender.com

When examining SNPs in DEGs, we identified reliable SNPs located in genes associated with immune response and oxidative stress. In the LB fish group, a missense mutation on *transmembrane protease serine 9-like (tmprss9)* (Fig. 6B) that causes the substitution of Histidine with Arginine in amino acid 22 (His22Arg) was present in all five analyzed individuals and absent in all five of the other phenotype. Moreover, among the isoforms of *dual oxidase 2-like (duox2) and dual oxidase maturation factor 1-like, (duoxa1)* (Fig. 8), we found up to 3 SNPs for each gene. Three missense mutations, including Ser267Gly, Ala740Asp, and Arg974Gln on *duox2* gene and three other missense mutations involving Leu55Phe, Ala213Thr, and Glu239Gly on *duoxa1* were present in the same four of the five low color-banded fish and absent in the other phenotype (Fig. 8).

## Discussion

In Atlantic salmon, flesh color is a complex qualitative trait, where its formation is influenced by several factors, including dietary formulations and absorption of carotenoid pigments, water and lipid content of flesh, as well as reproductive stage [34, 35]. Flesh discoloration in Atlantic salmon exposed to prolonged hot summers in Tasmania was recorded in recent years [36, 37] and is associated with reduced feed intake observed by the presence of feaces in the hindgut [38]. The number of starving fish peaks in March, and then declined over June to disappear when harvested in August [38]. This finding indicates that individuals drop in appeptite followed by starvation owing to the stress response during prolonged elevated temperature in summer. Grunenwald, Adams [20] pointed out that increased dietary astaxanthin, canthaxanthin and vitamin A did not prevent flesh discoloration. In optimal thermal conditions, temperature positively influences plasma Ax level and possibly controls the efficiency of Ax metabolism [39]. More recently, Wade, Clark [21] investigated the effects of a summer heatwave on farmed Atlantic salmon from two distinct groups cultured in sea cages. During that summer where the temperature was for 117 days above 18^o^C, flesh color was reduced initially in the front dorsal and then in the back central region. Furthermore, in autumn, a feeding and coloration recovery did not occur evenly among individuals. In the present study, we sampled fish from a single cage in June 2017. This time point was a few months past the heat stress of summer. From historical observations, while some fish at that site had recovered feeding and flesh color, others could not grow well and improve flesh colour at that time point. Therefore, we expected to find fish with different flesh color phenotypes. We hypothesized that the prolonged elevated temperature would not only affect flesh color, but also induce other changes in response to thermal stress, such as starvation and lipid metabolism. In which, these changes might no longer be recovered in some individuals. The flesh coloration variation in response to thermal stress, lipid metabolism and starvation were caused by molecular mechanisms that we studied at the gene expression level. Using both TruSeq and QuantSeq on the same subsets of HN and LB fish (n = 5 per group), a number of DEGs were detected in both phenotypes.

In the context of iron ion transport, *fertn-m* was upregulated in HN fish when compared to LB fish in our study. Iron is an essential trace element playing a key role as a biocatalyst or electron carrier for many biological reactions [40]. Because pathogens also require iron for proliferation and production of virulence factors, iron metabolism is closely related to the host innate immune response to prevent pathogens from iron uptake through an iron-withholding strategy [41, 42]. This strategy is controlled by the upregulation of positive acute-phase proteins, plasma proteins during acute phase response [40]. Ferritin is among these plasma proteins which segregate excess iron into a non-toxic and organic form for iron storage [43]. *fertn-m* function in Atlantic salmon was well described in previous studies [44]. *fertn-m* funtions in both mandatory iron storage stages of iron-oxidation and iron-mineralization [40, 45]. Several studies indicate that *fertn-m* is activated to respond to infections and oxidative stress in fish, including Atlantic salmon [40, 46–51]. In our previous studies, we showed that aerobic bacteria (*Pseudoalteromonadaceae*, *Vibrionaceae* and *Enterobacteriaceae* families), were enriched in the hindgut of low color fish. These bacterial families display enriched catabolic pathways which might impact on the host physiology leading to reduced flesh coloration [18, 52], suggesting competition over iron uptake between the host and aerobic bacteria.

Also, *catb* and *catl* were upregulated in HN fish in our present study. *catb* and *catl* are cysteine proteases involved in the self-defense mechanism against fish pathogens and the host immune defense in vertebrates [53, 54]. *catb* plays essential functions in pathological process and apoptosis [55]. *catb* may be associated with the regulation of follicular apoptosis in zebrafish [56] and affected the expression of CY genes in Japanese spiky sea cucumber [57]. They also activate the complex mechanism of fish proteolysis and muscle degradation to respond to diverse environmental and biological parameters [58]. LB fish downregulated *catl* and *catb* as compared to HN. This might indicate that LB fish could not activate the self-defense mechanism and respond to environmental conditions as efficiently as HN healthy fish.

Another group of genes (*alb2, fabp1, apoc1* and *apoa1*) related to lipid metabolism were also upregulated in HN fish. *alb2* is one of the most abundant proteins in the plasma, considered as the long-chain fatty acid transporter when it binds together to form complexes that circulate in the plasma [59, 60]. A radioactive labelling-based study proposed that Ax is associated with the serum lipoproteins, the primary transporters of esterified fatty acids and serum albumin [61]. We have found that *alb* is upregulated in the gut of HN fish and propose that this upregulation may increase the Ax absorption and transport from the gut into the blood system and then deposition in the muscle or liver. Due to their hydrophobic property, carotenoids or Ax are described to be closely associated with fatty acids and transported with them through the intestine and blood circulation. Moreover, *apoc1* was found to inhibit cholesteryl ester transferase protein activity that leads to increased accumulation of cholesterol in high-density lipoprotein (HDL) and a decrease in low-density lipoprotein (LDL) cholesterol level [62, 63]. Finally, upregulation of *apoa1* has been linked to increased accumulation of fatty acids and cholesterols into HDL [60, 63, 64]. HDL is known to have a major role in carrying fatty acids and carotenoids in Atlantic salmon [65, 66]. Thus, upregulation of *alb2, apoa1* and *apoc1* in HN fish implies that the uptake, absorption and metabolism of lipid or carotenoids occurs more intensively in HN fish while it might be partially impaired in LB fish.

The CYP450 enzyme family, together with important functions in the oxidation-reduction process, has also been known to induce stress response as a result of a variation in temperature [67]. In our study, *cyp2k1, cyp3a27* and *cyp1b1* were significantly upregulated in HN fish. Studies in birds indicated that CYP450 enzymes influence bird coloration in species such as zebra finches, red siskins and common canaries [68, 69]. CYP450 genes were also found to be upregulated in red flesh phenotype Chinook salmon when compared to white flesh phenotype [70]. In the latter study, the *CYP450 2J19* gene (*cyp2j19*), which is considered as a carotenoid ketolase and mediates red coloration in birds, was also upregulated in the pyloric caeca of red flesh phenotype Chinook salmon [70], although in the present study, *cyp2j19* was not a DEG. This finding suggests that *cyp2j19* might not be associated with the red coloration in the Atlantic salmon. However, the three CYP450 genes (*cyp2k1*, *cyp3a27* and *cyp1b1*) might have a link with flesh pigmentation in our study. Given the rapid evolutionary rate of CYP450s and their multiple roles across many metabolic processes [71, 72], it is plausible to assume that the three CYP450s identified as DEGs in our study neo-functionalized to have a role in carotenoid metabolism in Atlantic salmon and more comparative research across salmonids and other species with and without pigmentation should resolve this.

In our comparison, the neuropeptide *gastrin-releasing peptide (grp)* was upregulated in LB fish when compared with HN fish. Aside from functions in the gastrointestinal tract, some neuroendocrine peptides also impact on short-term food satiation and signal the brain to inhibit feeding [73]. In vertebrates, the process of food uptake and digestion is regulated and balanced by a series of endocrine events. In the gut-brain axis, neuroendocrine peptides are secreted by the stimulation of food intake available in the gastrointestinal tract [73, 74]. These peptides may act both through endocrine and neural systems. *grp* was previously shown to stimulate gastric acid release in the fish gut [73] while inhibiting gastric emptying and mediating satiety in fish [74]. In our current results, *grp* was upregulated in LB fish, which did not consume food well, while *grp* expression in HN fish was lower despite normal growth and feed intake. Some studies suggest that in mammals, the gastrin-releasing peptide incorporates a wide-ranging of physiological processes including stress responses, immune function, memory consolidation, and emotional behaviors [75, 76]. In rat, gastrin-releasing peptide levels was elevated and released in response to exposure to chronic stressor or a shock [77]. We proposee that *grp* may function as an anorexigenic signal in the gut-brain axis during prolonged thermal stress. The upregulation of *grp* in LB fish shows that this phenomenon might only occur when fish experienced chronic stress and starvation. This suggests that in LB fish, the control and balance of food intake with the feeding requirement was still dysfunctional likely due to persisting effects of the high thermal stress conditions during summer. Although the stress had been ceased, feeding activities in LB fish had still to recover completely. In addition, *grp* was considered as an islet neuropeptide that acts as a stimulator of insulin secretion induced by triggering of nervous system in mice [78]. This finding might apply to other vertebrates and be consistent with our result in the upregulation of *grp* in LB fish. We propose that *grp* could stimulate the secretion of insulin to balance energy metabolism in LB fish due to prolonged starvation. Accordingly, prolonged starvation is also the main reason for flesh discoloration, as fish likely lost a large amount of uptaken carotenoids to compensate for the amount used by physiological activities in the fish body.

When detecting whether there is a missense mutation in carotenoid-related genes, we found Pro21Thr missense mutation on *abcg8*, which highly expressed in HN fish. In humans and other mammals, *abcg8* and *abcg5* (*ATP-binding cassette, sub-family G (white), members 8 and 5*; also known as sterolins) are known to participate in the physiological pathways involving dietary cholesterol and non-cholesterol sterols [79]. These sterolins are necessary to secreting cholesterol efficiently into the bile and increase cholesterol levels in plasma and liver when the dietary cholesterol content changes [80, 81]. It has been shown that 2 % increase in dietary cholesterol improved Ax absorption and deposition as well as Ax levels in the plasma of Atlantic salmon [82]. Hence, in light of the aforementioned findings, additional cholesterol in Atlantic salmon diets could potentially result in increased Ax levels in the plasma, which would then be absorbed by the muscle, leading to a more intense flesh coloration. In addition, *abcg8* might have a critical function in increased carotenoid-binding by cholesterols in HN fish. With regard to to ATP-binding cassette, sub family, Zoric [83] reported that a missense mutation in *abcg2-1a*, resulting in the substitution of Asparagine with Serine in amino acid position 230 (Asn230Ser), is predicted to be associated with flesh color in Atlantic salmon because it is more prevalent in the pale than in the dark flesh phenotype. The complex *abcg5/g8* was previously associated with carotenoid metabolism and was predicted to function in limiting dietary carotenoid [84]. Therefore, in our study, it is possible that Pro21Thr missense mutation on *abcg8* plays an important role together with *abcg5* in the control of secreting carotenoid-binding cholesterols and absorption of dietary carotenoids and mobilization of carotenoids in Atlantic salmon.

In term of SNPs on DEGs identified in TruSeq, the six mutations on *duox2-l* and *duoxa1-l* that we detected in LB fish in the current study might potentially lead to the mismatched complex, which then affects their function of generating reactive oxygen species (ROS). Dual oxidase 2 (Duox2) is a member of the NADPH oxidase (NOX) family that generates reactive oxygen species (ROS). This enzyme catalyses the electron transfer from NAPDH-FAD to O_2_, generating superoxide (O_2_.^−^) or hydrogen peroxide (H_2_O_2_) by reducing molecular oxygen [85]. Duox1 and duox2 function solely in regulating specific maturation factors known as duox activators, Duoxa1 and Duoxa2, respectively [86, 87]. In a recent study, Duox and Duoxa proteins were found to form stable heterodimers and co-translocate to the plasma membrane (Fig. 8) [88]. Opposed to Nox1-5, Duox enzymes have an extended N-terminal extracellular domain known as peroxidase homology (PoxH) domain, followed by an additional transmembrane segment and an intracellular loop containing two calcium-binding EF-hand motifs (Fig. 8). There is a switch in ROS generation from H_2_O_2_ to O_2,_ which happens when Duox2 is mismatched with Duoxa1. Following that, the mismatched combination of Duox2 + Duoxa1 releases O_2_.^−^ together with H_2_O_2_, leading to the phenomenon of O_2_.^−^ leakage [89, 90]. In juvenile rainbow trout, dietary Ax supposedly enhanced the antioxidant defense system, which plays a role in the inactivation of ROS [91]. Consequently, we hypothesize that in LB fish, mutations which enhance ROS, together with a deficiency of Ax or carotenoids can be associated with an impaired antioxidant capability.

The qPCR validation confirms that the TruSeq data performance on genes with low expression was much more precise than the QuantSeq data. In addition, although the first ten genes in Fig. 7 (5 DEGs in both libraries and 5 DEGs in QuantSeq only, respectively) had high expression and the last five genes (5 DEGs in TruSeq only) had low expression in both QuantSeq and TruSeq data, the level of expression of the gene did not affect the RT-qPCR result. In RT-qPCR validation, it is evident that genes with high variation within biological replicates can not be considered as differentially expressed (*P > 0.05*) even though they were picked as DEGs and expressed highly in the QuantSeq and TruSeq data.

A comparison between TruSeq and QuantSeq on two subsets of distinct HN and LB flesh color phenotypes with five replicates revealed many DEGs in both the low and high sequencing depths. As a result, the sequencing analysis were compared to show the benefits and drawbacks of the two methods. In only QuantSeq with a low depth of sequencing, in the comparisons of 39 samples from 4 color phenotypes, there was a high variation in the number of normalized read counts within each group. A recent study of genetic and phenotypic correlations indicated that improvement in growth did not end in any flesh color variation in Atlantic salmon [35]. It suggested that flesh color might be affected by multiple genes and the response was not uniform. Accordingly, more replicates resulted in increased variation of gene expression between replicate samples and as a consequence, DEGs were hardly shown in our present study with increased sample size.

In the standard whole transcript method, mRNAs was fragmented before converted to cDNA. Therefore, the longer the transcript, the more fragments were created. On the other hand, 3’ mRNA method creates only one read for each transcript, so the read count reflects the level of gene expression. Bioinformatic analysis is also simplified since exon junction detection, and the normalization of reads to gene length are not required [31, 33]. As expected, TruSeq reads mapped transcripts evenly with a minor decline at the 5’ and 3’ end. This result is compatible with the finding from a previous study [30]. In contrast, QuantSeq reads covered primarily to the 3’ end. In vertebrates, genes can be alternatively spliced to create many distinct and expressed isoforms [33]. We hypothesize that TruSeq method is more suitable to determine differences in gene isoforms than QuantSEq. In addition, the proportion of read numbers mapped using TruSeq should increase with transcript size, while transcript size should not change the proportion of reads mapped to each transcript with QuantSEq. Therefore, only the TruSeq quantification values should be normalized to transcript length. It was therefore expected that the proportion between TruSeq and QuantSeq reads mapped per transcript should increase in favor of TruSeq reads numbers as the size of transcripts increases [31–33].

DEG analysis is one of main approaches of RNA-SEq. Following QuantSeq’s instruction, we loaded 96 QuantSeq libraries onto one lane to reduce sequencing cost and create about 2 million reads for each library. As per QuantSeq’s recommendation, it requires at least 10 million reads per library for QuantSeq transcriptomics in mammals [32]. Because the Atlantic salmon genome is smaller than those of mammals, we expected 2 million reads for each QuantSeq library would satisfy the recommendation. With TruSeq method, over 20 million reads for each library were generated to achieve the minimum requirement of DGE analysis. Therefore, TruSeq costs about 6 times more than QuantSeq per sample, however the cost/million reads is the same for either TruSeq or QuantSEq. Some studies have researched the effect of sequencing depth on RNA-Seq, in which a higher power was reached as sequencing depth rises (below 20–30 million reads) [92, 93]. Using DESq2 for differential expression analysis, we found that TruSeq detected more DEGs with low expression than QuantSEq. A previous study compared the efficiency of KAPPA whole transcript and Lexogen 3’RNA library preparation methods using the same depth of sequencing. This study also found that the whole transcript RNA-Seq method detected more DEGs, while more short transcripts was found using QuantSeq [30]. In human, a comparison of transcritompic analysis between Illumina TruSeq and QuantSeq in higher sequencing depth (30 million reads per TruSeq and 10 million reads per QuantSeq sample) was undertaken. Truseq again detected more DEGs then QuantSeq [32]. Moreover, when the sequencing depth of QuantSeq was elevated, the relationship between the TruSeq and QuantSeq results was strongly correlated at the gene expression levels [32]. This suggests that the depth of sequencing effects the number of detected DEGs, indicating that future salmon transcriptomics projects might benefit from having more than 2 million reads per library.

## Conclusions

In this study, we found that flesh pigmentation in Atlantic salmon is associated with lipid metabolism, specifically apolipoproteins, serum albumin and fatty acid-binding protein genes which are proposed to play important functions in the absorption, transport and deposition of carotenoids. Moreover, we speculate that *grp*, which is highly expressed in low banded fish, could inhibit their feeding behavior, leading to the dietary carotenoid shortage, resulting in the flesh discoloration in Atlantic salmon. Several SNPs in genes relating to carotenoid-binding cholesterol and oxidative stress were found in both flesh color phenotypes. Future studies are warranted to confirm if these SNPs have a strong relationship to flesh color, in which case they can be used to understand further the mechanisms involved in flesh color in Atlantic salmon and be tested for suitability as markers for selective breeding programs.

The comparison between the two gene expression studies showed that Lexogen QuantSeq is a cost-effective method of library preparation that targets known genes with high expression and no splicing variants. Due to its lower cost of library preparation, a higher number of samples could be analyzed, thus significantly reducing the number of false-positive DEGs detected. For longer transcripts with many isoforms or unknown genes, it is recommended to apply the Illumina TrueSeq method where lowly expressed genes and gene isoforms can be discovered.

## Materials and methods

### Experimental fish and sampling

In June 2017 (end of autumn), 50 fish (3.01 ± 0.09 kg, Mean ± SEM) were sampled randomly from a commercial population at a marine farm in Tasmania, Australia. The time point was chosen based on historical observations at that site showning that some fish can display flesh discoloration at different levels of severity as a result of elevetad summer temperatures. Fish were crowded in repeats utilizing a box-net routinely used for weight checks, and randomly sampled out of the crowd. Fish were euthanized through a non-recoverable percussive blow to the head using an automatic stunner, then put into an ice slurry container and immediately transported to the sampling site. For each fish, the hindgut was collected and preserved in RNAlater (Invitrogen, USA). Samples were kept overnight at 4^o^C before storing at -80^o^C until further analysis.

**Fig. 9:**
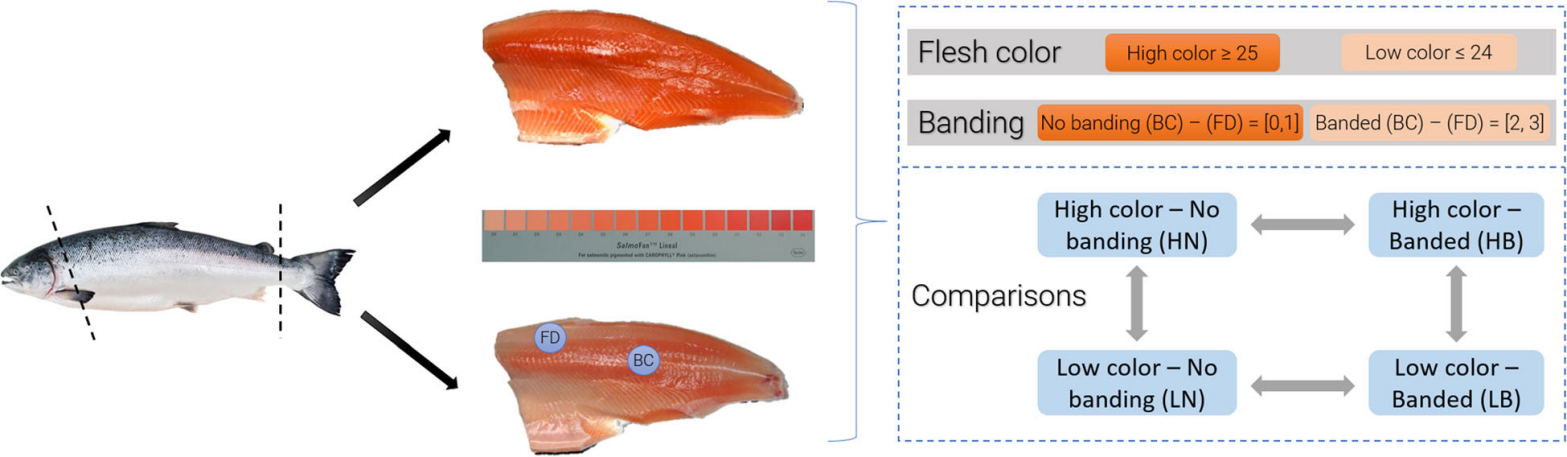
Flesh color assessment is based on the SalmoFan™ Lineal Card.

After sampling the hindgut, each fish had its flesh color visually assessed in standardized lighting conditions. After hand-filleting and trimming, the left fillet was color assessed and a score from 20 to 34 assigned to it by using the Roche SalmoFan™ Lineal Card (Hoffman-La Roche, Basel, Switzerland) (Fig. 9). To categorize localized discoloration, commercially known as “banding”, two regions were selected: the front dorsal region (FD; often affected by discoloration) and the back central (BC; which tends to retain color more evenly). The average score of color between the two regions was computed to classify as HIGH fillets when flesh color was higher than 24 on the SalmoFan or as LOW for flesh color equal or lower than 24. The severity of discoloration (banding) was based on the color score difference between the back central and the front dorsal region (severity = BC score – FD score) and it was classified as NONE for a difference from 0 to 1, or as BANDING from 2 to 3. Following the color assessment, 39 fis h were selected for analysis based on two criteria: average flesh color score and severity of banding; and divided into four groups: 13 high color-no banding (HN), 8 high color-banded (HB), 9 low color-no banding (LN) and 9 low color-banded (LB). There was no significant difference in Fulton’s condition factor (K) between the four groups (1.59, 1.69, 1.55 and 1.54 for HN, HB, LN and LB, respectively, *P > 0.05*). All fish work was carried out in compliance with the University of the Sunshine Coast (USC) animal ethics committee (AN/E/16/12). We confirm the study was carried out in compliance with the ARRIVE guidelines.

### RNA extraction, library preparation and sequencing

Total RNA was extracted from hindgut using RNeasy mini Kit (Qiagen, Cat no./ID: 74,104), treated with DNase I (RNase-free) (Biolabs, Cat no. M0303S) for gDNA removal, according to the manufacturer’s manual. The quantity, quality and integrity of the extracted RNA were assessed using NanoDrop 2000 spectrophotometer (ThermoFisher Scientific, USA) and Agilent 2100 Bioanalyzer (Agilent Technologies, Inc., USA). RNA samples used in the library preparation were chosen based on the following criteria: A260/280 ratio within 2.0-2.2, A260/A230 ratios > 1.8 and RIN (RNA Integrity Number) values > 9.

**Fig. 10:**
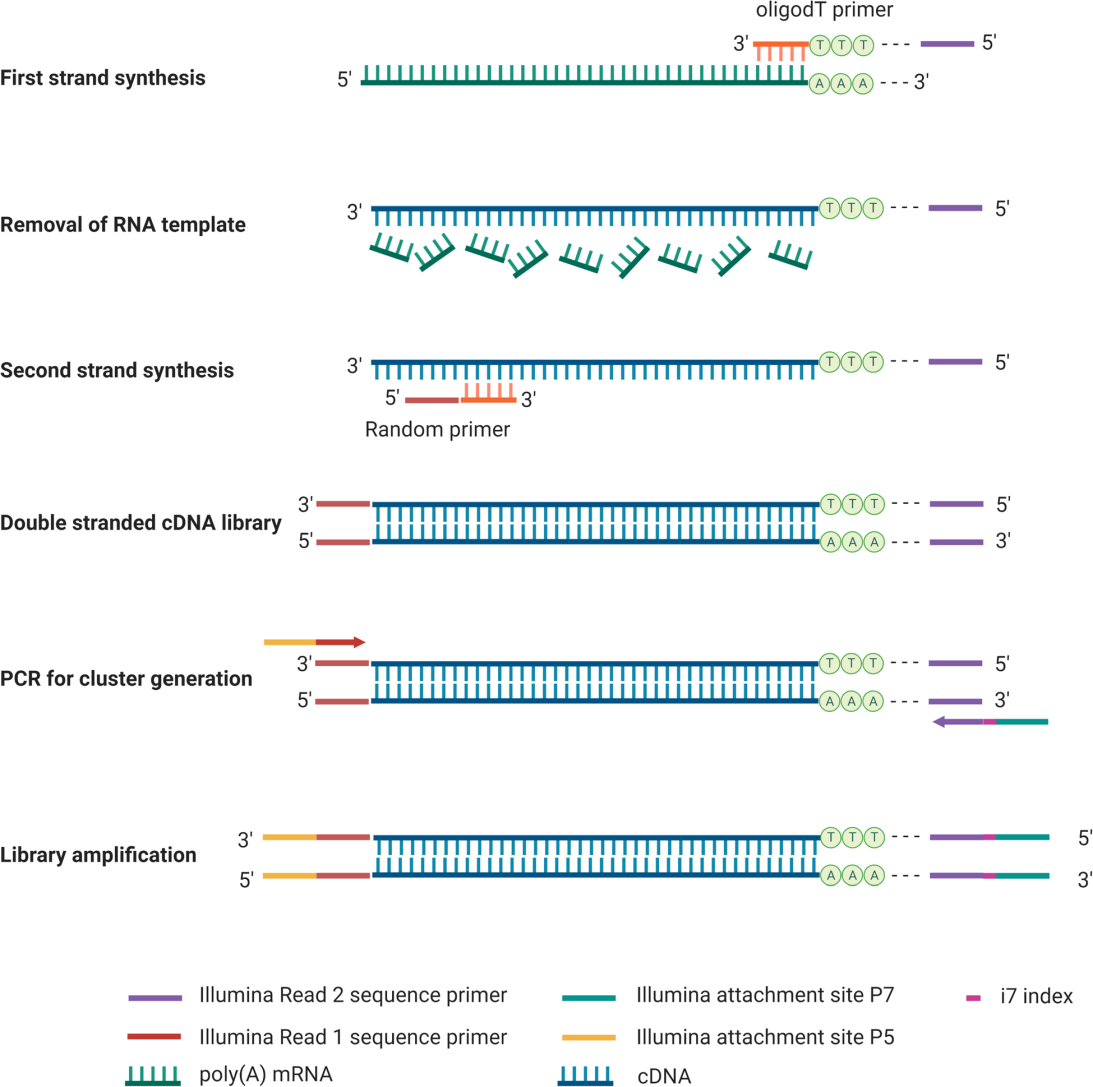
The workflow of QuantSeq 3’ mRNA library preparation. Created with BioRender.com

For QuantSeq library, cDNA synthesis and library preparation of 39 hindgut samples were performed using QuantSeq 3’ mRNA-Seq Library Kit for Illumina (FWD) (Lexogen, Cat no./ID: 015.96), Purification Module with Magnetic Beads (Lexogen, Cat no./ID: 022.96) and PCR Add-on Kit for Illumina (Lexogen, Cat no./ID: 020.96), according to the manufacturer’s instructions (Fig. 10). Briefly, the first cDNA strand is synthesized using an oligo (dT) primer containing the Illumina-specific Read 2 linker sequence (P7). A random primer containing the Illumina-specific Read 1 linker sequence (P5) and DNA polymerase were then used to synthesize the second cDNA strand. Paired-end sequencing is not recommended for QuantSeq library owing to the low quality of Read 2. Read 2 would begin with poly(T) stretch and sequence through the homopolymer stretch. Library quantity was measured using Quantus Fluorometer (Promega Corporation). Sequencing was done employing the Illumina HiSeq 2500 (100 bp, single-end) at the Australian Genome Research Facility.

A fish displaying HN was considered as an unaffected individual where flesh color is high and even, while a fish displaying LB as a heavily affected individual with abnormal flesh color. Therefore, given the difference between the two extreme phenotypes, HN and HB were predicted to have distinct genotypes. Therefore, in the comparison of the effectiveness of QuantSeq and TruSeq libraries, the same ten randomly chosen extracted RNA samples which were used for QuantSeq library were selected from two groups: five of extremely HN and five of extremely LB for repeated sequencing using Illumina library preparation. Extracted RNA samples were sent to Novogene (Hong Kong) for library preparation using Illumina TruSeq DNA PCR-Free Library Preparation kit, followed by sequencing on the Illumina HiSeq2500 (150 bp, pair-end reads).

The quality of the raw reads was examined using FastQC v.0.11.6 (http://www.bioinformatics.babraham.ac.uk/projects/fastqc/). For the QuantSeq dataset, sequencing adapter poly-A tail and low quality reads were trimmed using the BBDuk module in BBMap v.38.22 (–https://sourceforge.net/projects/bbmap/) with the following parameters “ref = polyA.fa.gz,truseq.fa.gz ftl = 8 k = 13 ktrim = r useshortkmers = t mink = 5 qtrim = r trimq = 10 minlength = 20”. For the TruSeq dataset, Trimmomatic [94] was used to remove low quality reads with some modifications: ILLUMINACLIP:TruSeq3-PE-2.fa:2:30:10 HEADCROP:8 LEADING:5 TRAILING:5. After trimming, trimmed reads from both datasets were aligned to the *S. salar* genome (Atlantic Salmon Genome - ICSASG_v2, https://www.ncbi.nlm.nih.gov/assembly/GCF_000233375.1/#/def) using HISAT2 v.2.1.0 with the default parameters [95]. Read counting was determined using HTSeq v.0.9.1 [96]. For each comparison, genes with less than 10 reads across all samples were discarded.

### Transcript coverage

Following mapping to the Atlantic salmon genome, all 20 BAM files from both TruSeq and QuantSeq data were loaded onto RSeQC v2.6.4 [97] to calculate transcript coverage. *SH2 domain-containing protein 4 A-like* (*sh24a*) gene coverage was visualized using two BAM files from TruSeq and QuantSeq of the same sample using Integrative Genomics Viewer [98].

### Distribution of mapped reads on different mature mRNA length

In QuantSeq, the number of mapped reads for each mature mRNA and mature mRNA length were scatter-plotted using ggplot2 package in RStudio 1.3.1093. In TruSeq, the number of mapped reads for each mature mRNA (RC) was normalized with mature mRNA length (L) following the formula below before scatter-plotting as done with QuantSeq.

$${NRC}_{i}=\frac{{RC}_{i}}{{L}_{i}}\times 1000 (normalized reads/ mature mRNA)$$

Where NRC_i_ = normalized reads count of mature mRNA i; RC_i_ = read counts of mature mRNA i and L_i_ = length of mature mRNA i (bp).

Mature mRNAs were firstly divided into eleven various groups based on their length at 1000 bp intervals (200–1000 bp, 1001–2000 bp and so on), with the last group being the total mature mRNA longer than 10,000 bp. A total number of mapped mature mRNA was then divided by the total number of mature mRNA in each group to identify the proportion of mRNA to which reads mapped from each library, at each length group.

### Differential expression analysis and gene ontology annotation

DEG analysis was conducted by DESeq2 in R (version 3.5.3) [99]. A gene was considered as DEG between two conditions when the following applied: |log_2_(fold change)| > 1, p-adjusted value < 0.05. The number of overlapping DEGs from the two datasets (TruSeq and QuantSeq datasets) was visualized using InveractiVenn [100].

All DEGs were then loaded into OmicsBox v1.4 (*OmicsBox – Bioinformatics Made Easy, BioBam Bioinformatics, March 3, 2019*, https://www.biobam.com/omicsbox*)* for further downstream analysis. Here, DEGs were searched against the NCBI non-redundant database (nr) using BlastX algorithm with E-value threshold set at 1.00E^-6^. Blasted transcripts were afterwards annotated then classified into three ontology categories: molecular function, cellular component and biological process. The distribution of gene onthology (GO) terms from two different libraries was subsequently plotted using Web Gene Ontology Annotation Plot v.2.0 (WEGO 2.0) software [101].

### Real-time quantitative PCR

Fifteen genes were chosen from three clusters based on strong significance of Log2FoldChange, regardless of their function: five DEGs in both library methods; five DEGs only in QuantSeq library; and five genes only in TruSeq library. 18 S rRNA was used as a housekeeping gene (HKG). Sixteen pairs of primers were designed using the ‘Assay Design Center’ available at the Roche website (https://qpcr.probefinder.com/organism.jsp) and listed in Table 2. Real-time quantitative polymerase chain reaction (RT-qPCR) was performed as previously described [102] with slight modifications to validate DEGs found in either or both sequencing datasets. cDNA templates were synthesized using Bioline Tetro cDNA Synthesis kit (Cat. No. BIO-65,043) from the same RNA samples used for sequencing. Primers were mixed with the cDNA and FastStart Universal Probe Master (Rox; Roche Diagnostics GmbH) and Universal ProbeLibrary Probe (Roche). The HKG 18 S rRNA served to normalize the quantification. qPCR cycles included 10 min incubation at 94 °C, followed by 40 cycles of 94 °C for 10 s and 60 °C for 30 s, with green fluorescence measurement on each cycle at 60 °C. Reactions were performed in Rotor-Gene Q (Qiagen). Relative quantification was calculated by equilibrating to the level of HKG per sample and the sample with the lowest value (2^−∆∆CT^). Statistical analysis of the resulting RQs was performed using ANOVA, followed by Wilcoxon test, with *P < 0.05* considered as statistically significant.

**Table. 2:** Sixteen DEGs selected for validation with RT-qPCR

Gene ID	DEG Group	Forward primer 5’-3’	Reverse primer 5’-3’
*(mfi2) antigen p97 (melanoma associated)*	QuantSeq only	cagcagggaaaagctacgg	tcactgtcccctgtgtagctc
*(epdm) ependymin-like*	QuantSeq only	aaccacacaatgcaagccta	agctcccaacagccagatac
*(lyama) lysosomal alpha-mannosidase*	QuantSeq only	aacagctacctgcagacgtg	tttccttgagatgggaccac
*(fabp1) fatty acid binding protein 1, liver*	QuantSeq only	gggcaccaaggtcatagtca	tcttctctccagtcaaagtctcg
*(fertn-m) ferritin, middle subunit-like*	QuantSeq only	cgtgatgagtggggcaat	cagggcctggttcacatt
*(s2p4a-l) SH2 domain-containing protein 4 A-like*	TruSeq only	gcaacagcacagacgaccta	atgttgctgtggtgggttct
*(noxo1) NADPH oxidase organizer 1-like*	TruSeq only	atgggctggaggacatga	gctcacaaaagggtgtgtca
*(fclt3) fucolectin-3-like*	TruSeq only	atgagggcattcacaacaca	tccttccacacctgatgtcc
*(tetn) tetranectin*	TruSeq only	gtcagtggtgtgcgtttgtt	tctgttggaatgtggattgc
*(ovcm2) ovochymase-2-like*	TruSeq only	cgtttcctcagcaaccaag	gggatcggtggtccagtaa
*(ldltn) ladderlectin-like*	Both libraries	ctttgtgtcgccctctctg	gacattgttgagtatttggctgtc
*(cd209) CD209 antigen-like*	Both libraries	tggtcacaattaaataatgttttgg	gaatgtttatttagtcatgctggtg
*(es1p) ES1 protein homolog, mitochondrial-like*	Both libraries	agttgacttccagttacactacaacag	gcagtctgggtcgtcttctc
*(umy1b) unconventional myosin-Ib-like*	Both libraries	aggaatgccatgcagattgt	accagctccagaaccgact
*(glfsy) GDP-L-fucose synthase*	Both libraries	ctgattggctgtcaagcaac	gaaggtttgactccccatga
*(18 S) 18 S ribosomal RNA*	House keeping gene	aggactccggttctattttgtg	cggccgtccctcttaatc

### Single nucleotide-polymorphisms (SNPs) analysis

From the Illumina TruSeq data, two sets of variants were detected with FreeBayes [103], a Bayesian genetic variant detector designed to find polymorphisms, specifically SNPs. All SNPs were annotated and the effects of the SNP variants on genes and proteins were determined with Ensembl Variant Effect Predictor (VEP) [104]. All missense mutations from each group were merged using BCFTools [105], a set of utilities that manipulate variant calls then used to compare and create a list of unique SNPs from each group.

## Supplementary Information

**Additional file 1: Supplementary 1.** Distribution of GO terms and the most significant GO terms in comparison of TruSeq and QuantSeq library.

**Additional file 2: Supplementary 2.** Differentially expressed genes in QuantSeq and TruSeq data.

## Data Availability

The datasets supporting the conclusions of this article are available in the BioProject Accession number PRJNA706530, ID 706,530 - BioProject - NCBI (nih.gov).

## Abbreviations

*abcg5*: *ATP-binding cassette, sub-family G (white), member 5*
*abcg8*: *ATP-binding cassette, sub-family G (white), member 8*
*alb2*: *Serum albumin 2*
*apoa1*: *Apolipoprotein A-I*
*apoc1*: *Apolipoprotein C-I*
Ax: Astaxanthin
BC: Back central region
*catb*: *Cathepsin B*
*catl1*: *Cathepsin L1-like*
CY: cytochrome
*cyp1b1*: *CYP450 1B1-like*
*cyp2j19*: *CYP450 2J19*
*cyp2k1*: *CYP450 2K1-like*
*cyp3a27*: *CYP450 3A27-like*
*cypc6a*: *Cytochrome c oxidative subunit 6 A mitochondrial-like*
DEGs: Differentially expressed genes
*duox2*: *Dual oxidase 2-like*
*duoxa1*: *Dual oxidase maturation factor 1-like*
*dabp1*: *Fatty acid-binding protein 1, liver*
FD: Front dorsal region
*fertn-m*: *Ferritin middle subunit*
*grp*: *Gastrin-releasing peptide*
HB: High-banded
HDL: High-density lipoprotein
HN: High-no banding
LB: Low-banded
LDL: Low-density lipoprotein
LN: Low-no banding
NGS: Next-generation sequencing
QuantSeq: Lexogen’s QuantSeq 3’ mRNA library preparation
RIN: RNA Integrity Number
RNA-Seq: RNA sequencing
ROS: Reactive oxygen species
*sh24a*: *SH2 domain-containing protein 4 A-like*
SNPs: Single nucleotide polymorphisms
*tmprss9*: *Transmembrane protease serine 9-like*
TruSeq: Illumina’s TruSeq kit
VEP: Ensembl Variant Effect Predictor

## References

[1] Anderson S. Salmon Color and the Consumer. Microbehavior and Macroresults: Proceedings of the Tenth Biennial Conference of the International Institute ofFisheries Economics and Trade, July 10-14, 2000, Corvallis, Oregon, USA.Compiled by Richard S. Johnston and Ann L. Shriver. Corvallis: InternationalInstitute of Fisheries Economics and Trade (IIFET); 2001.

[2] Chitchumroonchokchai C, Failla ML (2017). Bioaccessibility and intestinal cell uptake of astaxanthin from salmon and commercial supplements. Food Research International.

[3] Olsen RE, Kiessling A, Milley JE, Ross NW, Lall SP (2005). Effect of lipid source and bile salts in diet of Atlantic salmon, Salmo salar L., on astaxanthin blood levels. Aquaculture.

[4] Nickell DC, Bromage NR (1998). The effect of dietary lipid level on variation of flesh pigmentation in rainbow trout (Oncorhynchus mykiss). Aquaculture.

[5] Bjerkeng B, Hamre K, Hatlen B, Wathne E (2001). Astaxanthin deposition in fillets of Atlantic salmon Salmo salar L. fed two dietary levels of astaxanthin in combination with three levels of α-tocopheryl acetate. Aquac Res.

[6] Bjerkeng B, Hatlen B, Wathne E (1999). Deposition of astaxanthin in fillets of Atlantic salmon (Salmo salar) fed diets with herring, capelin, sandeel, or Peruvian high PUFA oils. Aquaculture.

[7] Elliott JM, Elliott JA (2010). Temperature requirements of Atlantic salmon Salmo salar, brown trout Salmo trutta and Arctic charr Salvelinus alpinus: predicting the effects of climate change. Journal of Fish Biology.

[8] Hevrøy EM, Waagbø R, Torstensen BE, Takle H, Stubhaug I, Jørgensen SM (2012). Ghrelin is involved in voluntary anorexia in Atlantic salmon raised at elevated sea temperatures. Gen Comp Endocrinol.

[9] Oppedal F, Dempster T, Stien LH (2011). Environmental drivers of Atlantic salmon behaviour in sea-cages: A review. Aquaculture.

[10] Johansson D, Ruohonen K, Kiessling A, Oppedal F, Stiansen J-E, Kelly M (2006). Effect of environmental factors on swimming depth preferences of Atlantic salmon (Salmo salar L.) and temporal and spatial variations in oxygen levels in sea cages at a fjord site. Aquaculture.

[11] Stehfest KM, Carter CG, McAllister JD, Ross JD, Semmens JM (2017). Response of Atlantic salmon Salmo salar to temperature and dissolved oxygen extremes established using animal-borne environmental sensors. Sci Rep.

[12] Hevrøy EM, Waagbø R, Torstensen BE, Takle H, Stubhaug I, Jørgensen SM, Torgersen T, Tvenning L, Susort S, Breck O, Hansen T. Ghrelin is involved in voluntary anorexia in Atlantic salmon raised at elevated sea temperatures. Gen Comp Endocrinol. 2012;175(1):118-34. 10.1016/j.ygcen.2011.10.007. Epub 2011 Oct 21. 10.1016/j.ygcen.2011.10.007 22036890

[13] Barton B. General biology of salmonids. Principles of Salmonid Aquaculture. W. Pennell and B. Barton. Amsterdam: Elsevier; 1996. pp 29-95.

[14] Vikeså V, Nankervis L, Hevrøy EM (2017). Appetite, metabolism and growth regulation in Atlantic salmon (Salmo salar L.) exposed to hypoxia at elevated seawater temperature. Aquac Res.

[15] Lund SG, Caissie D, Cunjak RA, Vijayan MM, Tufts BL (2002). The effects of environmental heat stress on heat-shock mRNA and protein expression in Miramichi Atlantic salmon (Salmo salar) parr. Canadian Journal of Fisheries and Aquatic Sciences.

[16] Olsvik PA, Vikeså V, Lie KK, et al. Transcriptional responses to temperature and low oxygen stress in Atlantic salmon studied with next-generation sequencing technology. BMC Genomics. 2013;14:817. 10.1186/1471-2164-14-817. 10.1186/1471-2164-14-817 PMC4046827 24261939

[17] Pankhurst NW, King HR (2010). Temperature and salmonid reproduction: implications for aquaculture. Journal of Fish Biology.

[18] Nguyen CDH, Amoroso G, Ventura T, Elizur A. Assessing the Pyloric Caeca and Distal Gut Microbiota Correlation with Flesh Color in Atlantic Salmon (*Salmo salar* L., 1758). Microorganisms. 2020;8:1244. 10.3390/microorganisms8081244. 10.3390/microorganisms8081244 PMC7464769 32824332

[19] Nakano T, Kameda M, Shoji Y, Hayashi S, Yamaguchi T, Sato M (2014). Effect of severe environmental thermal stress on redox state in salmon. Redox Biology.

[20] Grunenwald M, Adams MB, Carter CG, Nichols DS, Koppe W, Adams LR. Pigment depletion in Atlantic salmon (*Salmo salar*) starved at high temperature: effect of dietary carotenoid type and vitamin E level, Proceedings of the 17th International Symposium on Feeding and Nutrition of Fish, 05-10 June 2016, Idaho, USA. 2016. [Conference Extract]

[21] Wade NM, et al. "Effects of an unprecedented summer heatwave on the growth performance, flesh colour and plasma biochemistry of marine cage-farmed Atlantic salmon (Salmo salar)." J Therm Biol. 2019;80:64–74. 10.1016/j.jtherbio.2018.12.021 30784489

[22] Lister R, O’Malley RC, Tonti-Filippini J, Gregory BD, Berry CC, Millar AH, et al. Highly Integrated Single-Base Resolution Maps of the Epigenome in Arabidopsis. Cell. 2008;133(3):523–36. 10.1016/j.cell.2008.03.029 PMC2723732 18423832

[23] Emrich SJ, Barbazuk WB, Li L, Schnable PS (2007). Gene discovery and annotation using LCM-454 transcriptome sequencing. Genome research.

[24] Kumar R, Ichihashi Y, Kimura S, Chitwood D, Headland L, Peng J (2012). A High-Throughput Method for Illumina RNA-Seq Library Preparation. Frontiers in Plant Science.

[25] Amoroso G, Ventura T, Cobcroft JM, Adams MB, Elizur A, Carter CG (2016). Multigenic Delineation of Lower Jaw Deformity in Triploid Atlantic Salmon (Salmo salar L.). PLoS ONE.

[26] Ozsolak F, Milos PM (2011). RNA sequencing: advances, challenges and opportunities. Nature Reviews Genetics.

[27] Park Y-S, Kim S, Park D-G, Kim DH, Yoon K-W, Shin W (2019). Comparison of library construction kits for mRNA sequencing in the Illumina platform. Genes & Genomics.

[28] Lahens NF, Ricciotti E, Smirnova O, Toorens E, Kim EJ, Baruzzo G (2017). A comparison of Illumina and Ion Torrent sequencing platforms in the context of differential gene expression. BMC Genomics.

[29] Wilhelm BT, Marguerat S, Goodhead I, Bähler J (2010). Defining transcribed regions using RNA-sEq. Nature Protocols.

[30] Ma F, Fuqua BK, Hasin Y, Yukhtman C, Vulpe CD, Lusis AJ (2019). A comparison between whole transcript and 3’ RNA sequencing methods using Kapa and Lexogen library preparation methods. BMC Genomics.

[31] Moll P, Ante M, Seitz A, Reda T (2014). QuantSeq 3′ mRNA sequencing for RNA quantification. Nature Methods.

[32] Corley SM, Troy NM, Bosco A, Wilkins MR (2019). QuantSEq. 3′ Sequencing combined with Salmon provides a fast, reliable approach for high throughput RNA expression analysis. Sci Rep.

[33] Stark R, Grzelak M, Hadfield J (2019). RNA sequencing: the teenage years. Nature Reviews Genetics.

[34] Norris AT, Cunningham EP (2004). Estimates of phenotypic and genetic parameters for flesh colour traits in farmed Atlantic salmon based on multiple trait animal model. Livestock Production Science.

[35] Garber AF, Amini F, Gezan SA, Swift BD, Hodkinson SE, Nickerson J (2019). Genetic and phenotypic evaluation of harvest traits from a comprehensive commercial Atlantic salmon, Salmo salar L., broodstock program. Aquaculture.

[36] Grünenwald M, Adams MB, Carter CG, Nichols DS, Koppe W, Verlhac-Trichet V (2019). Pigment-depletion in Atlantic salmon (Salmo salar) post-smolt starved at elevated temperature is not influenced by dietary carotenoid type and increasing α-tocopherol level. Food Chemistry.

[37] Grünenwald M, Carter CG, Nichols DS, Adams MB, Adams LR (2020). Heterogeneous astaxanthin distribution in the fillet of Atlantic salmon post-smolt at elevated temperature is not affected by dietary fatty acid composition, metabolic conversion of astaxanthin to idoxanthin, or oxidative stress. Aquaculture.

[38] Amoroso G, Nguyen C.D.H, Vo T.T.M, Ventura T, Elizur A. Understanding flesh colour variation in Atlantic salmon: molecular mechanisms and genetic effect. University of the Sunshine Coast (USC); 2020. Report No.: FRDC Project No 2014 – 248 Contract No.: Final Report ISBN: 978-1-925476-12-5.

[39] Ytrestøyl T, Struksnæs G, Koppe W, Bjerkeng B (2005). Effects of temperature and feed intake on astaxanthin digestibility and metabolism in Atlantic salmon, Salmo salar. Comparative Biochemistry and Physiology - B Biochemistry and Molecular Biology.

[40] Lee J-H, Pooley NJ, Mohd-Adnan A, Martin SAM (2014). Cloning and characterisation of multiple ferritin isoforms in the Atlantic salmon (Salmo salar). PLOS ONE.

[41] Posey JE, Gherardini FC (2000). Lack of a role for iron in the lyme disease pathogen. Science.

[42] Ong ST, Shan Ho JZ, Ho B, Ding JL (2006). Iron-withholding strategy in innate immunity. Immunobiology.

[43] Arosio P, Levi S (2010). Cytosolic and mitochondrial ferritins in the regulation of cellular iron homeostasis and oxidative damage. Biochimica et Biophysica Acta (BBA) -. General Subjects.

[44] Andersen O, Dehli A, Standal H, Giskegjerde TA, Karstensen R, Rørvik KA (1995). Two ferritin subunits of Atlantic salmon (Salmo salar): cloning of the liver cDNAs and antibody preparation. Mol Mar Biol Biotechnol.

[45] Mignogna G, Chiaraluce R, Consalvi V, Cavallo S, Stefanini S, Chiancone E (2002). Ferritin from the spleen of the Antarctic teleost Trematomus bernacchii is an M-type homopolymer. European Journal of Biochemistry.

[46] LeBlanc F, Laflamme M, Gagné N (2010). Genetic markers of the immune response of Atlantic salmon (Salmo salar) to infectious salmon anemia virus (ISAV). Fish & Shellfish Immunology.

[47] Robertson LS, McCormick SD (2012). The effect of nonylphenol on gene expression in Atlantic salmon smolts. Aquatic Toxicology.

[48] Peatman E, Baoprasertkul P, Terhune J, Xu P, Nandi S, Kucuktas H (2007). Expression analysis of the acute phase response in channel catfish (Ictalurus punctatus) after infection with a Gram-negative bacterium. Developmental & Comparative Immunology.

[49] Neves JV, Wilson JM, Rodrigues PNS (2009). Transferrin and ferritin response to bacterial infection: The role of the liver and brain in fish. Developmental & Comparative Immunology.

[50] Wang W, Zhang M, Sun L (2011). Ferritin M of Cynoglossus semilaevis: An iron-binding protein and a broad-spectrum antimicrobial that depends on the integrity of the ferroxidase center and nucleation center for biological activity. Fish & Shellfish Immunology.

[51] Zheng, W.-j., et al. "Identification and analysis of a Scophthalmus maximus ferritin that is regulated at transcription level by oxidative stress and bacterial infection." Comparative Biochemistry and Physiology Part B. Biochem Mol Biol Educ. 2010;156(3):222–8. 10.1016/j.cbpb.2010.03.012 20382253

[52] Nguyen CDH, Amoroso G, Ventura T, Minich JJ, Elizur A (2020). Atlantic Salmon (Salmo salar L., 1758) Gut Microbiota Profile Correlates with Flesh Pigmentation: Cause or Effect?. Marine Biotechnology.

[53] Subramanian S, MacKinnon SL, Ross NW (2007). A comparative study on innate immune parameters in the epidermal mucus of various fish species. Comparative Biochemistry and Physiology Part B: Biochemistry and Molecular Biology.

[54] Zhou Z-j, Qiu R, Zhang J (2015). Molecular characterization of the cathepsin B of turbot (Scophthalmus maximus). Fish Physiology and Biochemistry.

[55] Liang F-R, He H-S, Zhang C-W, Xu X-M, Zeng Z-P, Yuan J-P (2018). Molecular cloning and functional characterization of cathepsin B from Nile tilapia (Oreochromis niloticus). International Journal of Biological Macromolecules.

[56] Eykelbosh AJ, Van Der Kraak G (2010). A role for the lysosomal protease cathepsin B in zebrafish follicular apoptosis. Comparative Biochemistry and Physiology Part A: Molecular & Integrative Physiology.

[57] Chen H, Lv M, Lv Z, Li C, Zhang W, Zhao X (2017). Divergent roles of three cytochrome c in CTSB-modulating coelomocyte apoptosis in Apostichopus japonicus. Developmental & Comparative Immunology.

[58] Gaarder MØ, Bahuaud D, Veiseth-Kent E, Mørkøre T, Thomassen MS (2012). Relevance of calpain and calpastatin activity for texture in super-chilled and ice-stored Atlantic salmon (Salmo salar L.) fillets. Food Chemistry.

[59] Trigatti BL, Gerber GE (1995). A direct role for serum albumin in the cellular uptake of long-chain fatty acids. Biochemical Journal.

[60] Fasano M, Curry S, Terreno E, Galliano M, Fanali G, Narciso P (2008). The extraordinary ligand binding properties of human serum albumin. IUBMB Life.

[61] Aas GH, Bjerkeng B, Storebakken T, Ruyter B (1999). Blood appearance, metabolic transformation and plasma transport proteins of 14 C-astaxanthin in Atlantic salmon (Salmo salar L.). Fish Physiology and Biochemistry.

[62] Dumont L, Gautier T, de Barros J-PP, Laplanche H, Blache D, Ducoroy P (2005). Molecular Mechanism of the Blockade of Plasma Cholesteryl Ester Transfer Protein by Its Physiological Inhibitor Apolipoprotein CI. Journal of Biological Chemistry.

[63] Feingold KR. Introduction to Lipids and Lipoproteins. [Updated 2021 Jan 19]. In: Feingold KR, Anawalt B, Boyce A, et al., editors. Endotext [Internet]. South Dartmouth (MA): MDText.com, Inc.; 2000. Available from: https://www.ncbi.nlm.nih.gov/books/NBK305896/.

[64] Trigatti BL, Gerber GE (1995). A direct role for serum albumin in the cellular uptake of long-chain fatty acids. Biochemical Journal.

[65] Ando S, Takeyama T, Hatano M (1986). Transport Associated with Serum Vitellogenin of Carotenoid in Chum Salmon (Oncorhynchus keta). Agricultural and Biological Chemistry.

[66] March BE, Hajen WE, Deacon G, MacMillan C, Walsh MG (1990). Intestinal absorption of astaxanthin, plasma astaxanthin concentration, body weight, and metabolic rate as determinants of flesh pigmentation in salmonid fish. Aquaculture.

[67] Andersson T, Förlin L (1992). Regulation of the cytochrome P450 enzyme system in fish. Aquat Toxicol.

[68] Lopes Ricardo J, Johnson James D, Toomey Matthew B, Ferreira Mafalda S, Araujo Pedro M, Melo-Ferreira J (2016). Genetic basis for red coloration in birds. Current Biology.

[69] Mundy Nicholas I, Stapley J, Bennison C, Tucker R, Twyman H, Kim K-W (2016). Red carotenoid coloration in the zebra finch Is controlled by a cytochrome P450 gene cluster. Current Biology.

[70] Madaro A, Torrissen O, Whatmore P, Lall SP, Schmeisser J, Trichet VV (2020). Red and White Chinook Salmon (Oncorhynchus tshawytscha): Differences in the Transcriptome Profile of Muscle, Liver, and Pylorus. Mar Biotechnol.

[71] Zanger UM, Schwab M (2013). Cytochrome P450 enzymes in drug metabolism: Regulation of gene expression, enzyme activities, and impact of genetic variation. Pharmacology & Therapeutics.

[72] Burkina V, Zlabek V, Zamaratskaia G (2015). Effects of pharmaceuticals present in aquatic environment on Phase I metabolism in fish. Environmental Toxicology and Pharmacology.

[73] Jönsson E, Forsman A, Einarsdottir IE, Egnér B, Ruohonen K, Thrandur Björnsson B (2006). Circulating levels of cholecystokinin and gastrin-releasing peptide in rainbow trout fed different diets. Gen Comp Endocrinol.

[74] Xu M, Volkoff H (2009). Molecular characterization of ghrelin and gastrin-releasing peptide in Atlantic cod (Gadus morhua): Cloning, localization, developmental profile and role in food intake regulation. Gen Comp Endocrinol.

[75] Roesler R, Schwartsmann G (2012). Gastrin-releasing peptide receptors in the central nervous system: role in brain function and as a drug target. Frontiers in Endocrinology.

[76] Roesler R, Kent P, Luft T, Schwartsmann G, Merali Z (2014). Gastrin-releasing peptide receptor signaling in the integration of stress and memory. Neurobiology of Learning and Memory.

[77] Merali Z, Kent P, Anisman H (2002). Role of bombesin-related peptides in the mediation or integration of the stress response. Cellular and Molecular Life Sciences CMLS.

[78] Persson K, Pacini G, Sundler F, Ahrén B (2002). Islet Function Phenotype in Gastrin-Releasing Peptide Receptor Gene-Deficient Mice. Endocrinology.

[79] Hazard SE, Patel SB (2007). Sterolins ABCG5 and ABCG8: regulators of whole body dietary sterols. Pflügers Archiv - European Journal of Physiology.

[80] Yu L, Hammer RE, Li-Hawkins J, Von Bergmann K, Lutjohann D, Cohen JC, Hobbs HH. Disruption of Abcg5 and Abcg8 in mice reveals their crucial role in biliary cholesterol secretion. Proc Natl Acad Sci USA. 2002;99(25):16237–42. 10.1073/pnas.252582399. Epub 2002 Nov 20. 10.1073/pnas.252582399 PMC138595 12444248

[81] Kortner TM, Gu J, Krogdahl A, Bakke AM (2013). Transcriptional regulation of cholesterol and bile acid metabolism after dietary soyabean meal treatment in Atlantic salmon (Salmo salar L.). Br J Nutr.

[82] Chimsung N, Tantikitti C, Milley Joyce E, Verlhac-Trichet V, Lall Santosh P (2012). Effects of various dietary factors on astaxanthin absorption in Atlantic salmon (Salmo salar). Aquac Res.

[83] Zoric N (2017). Characterization of genes and gene products influencing carotenoid metabolism in Atlantic salmon.

[84] Herron KL, McGrane MM, Waters D, Lofgren IE, Clark RM, Ordovas JM (2006). The ABCG5 Polymorphism Contributes to Individual Responses to Dietary Cholesterol and Carotenoids in Eggs. The Journal of Nutrition.

[85] De Deken X, Corvilain B, Dumont JE, Miot F (2013). Roles of DUOX-Mediated Hydrogen Peroxide in Metabolism, Host Defense, and Signaling. Antioxidants & Redox Signaling.

[86] Hulur I, Hermanns P, Nestoris C, Heger S, Refetoff S, Pohlenz J (2011). A Single Copy of the Recently Identified Dual Oxidase Maturation Factor (DUOXA) 1 Gene Produces Only Mild Transient Hypothyroidism in a Patient with a Novel Biallelic DUOXA2 Mutation and Monoallelic DUOXA1 Deletion. The Journal of Clinical Endocrinology & Metabolism.

[87] Ueyama T, Sakuma M, Ninoyu Y, Hamada T, Dupuy C, Geiszt M (2015). The extracellular A-loop of dual oxidases affects the specificity of reactive oxygen species release. The Journal of biological chemistry.

[88] Drevet S, Gavazzi G, Grange L, Dupuy C, Lardy B (2018). Reactive oxygen species and NADPH oxidase 4 involvement in osteoarthritis. Experimental Gerontology.

[89] Zamproni I, Grasberger H, Cortinovis F, Vigone MC, Chiumello G, Mora S (2008). Biallelic Inactivation of the Dual Oxidase Maturation Factor 2 (DUOXA2) Gene as a Novel Cause of Congenital Hypothyroidism. The Journal of Clinical Endocrinology & Metabolism.

[90] Morand S, Ueyama T, Tsujibe S, Saito N, Korzeniowska A, Leto TL (2008). Duox maturation factors form cell surface complexes with Duox affecting the specificity of reactive oxygen species generation. The FASEB Journal.

[91] Rahman MM, Khosravi S, Chang KH, Lee S-M (2016). Effects of dietary inclusion of astaxanthin on growth, muscle pigmentation and antioxidant capacity of juvenile rainbow trout (Oncorhynchus mykiss). Prev Nutr Food Sci.

[92] Liu Y, Zhou J, White KP (2014). RNA-seq differential expression studies: more sequence or more replication?. Bioinformatics.

[93] Ching T, Huang S, Garmire LX (2014). Power analysis and sample size estimation for RNA-Seq differential expression. RNA.

[94] Bolger AM, Lohse M, Usadel B. Trimmomatic: a flexible trimmer for Illumina sequence data. Bioinformatics. 2014;30(15):2114-20. 10.1093/bioinformatics/btu170. Epub 2014 Apr 1. 10.1093/bioinformatics/btu170 PMC4103590 24695404

[95] Kim D, Langmead B, Salzberg SL (2015). HISAT: a fast spliced aligner with low memory requirements. Nature Methods.

[96] Anders S, Pyl PT, Huber W (2015). HTSeq–a Python framework to work with high-throughput sequencing data. Bioinformatics (Oxford, England).

[97] Wang L, Wang S, Li W (2012). RSeQC: quality control of RNA-seq experiments. Bioinformatics.

[98] Robinson JT, Thorvaldsdóttir H, Winckler W, Guttman M, Lander ES, Getz G (2011). Integrative genomics viewer. Nat Biotechnol.

[99] Love MI, Huber W, Anders S (2014). Moderated estimation of fold change and dispersion for RNA-seq data with DESeq2. Genome Biology.

[100] Heberle H, Meirelles GV, da Silva FR, Telles GP, Minghim R (2015). InteractiVenn: a web-based tool for the analysis of sets through Venn diagrams. BMC Bioinform.

[101] Ye J, Zhang Y, Cui H, Liu J, Wu Y, Cheng Y (2018). WEGO 2.0: a web tool for analyzing and plotting GO annotations, 2018 update. Nucleic Acids Research.

[102] Ventura T, Fitzgibbon Q, Battaglene S, Sagi A, Elizur A (2015). Identification and characterization of androgenic gland specific insulin-like peptide-encoding transcripts in two spiny lobster species: Sagmariasus verreauxi and Jasus edwardsii. Gen Comp Endocrinol.

[103] Garrison, Erik & Marth, Gabor. Haplotype-based variant detection from short-read sequencing. arXiv. 2012;1207.

[104] McLaren W, Gil L, Hunt SE, Riat HS, Ritchie GRS, Thormann A (2016). The Ensembl Variant Effect Predictor. Genome Biology.

[105] Danecek P, McCarthy SA (2017). BCFtools/csq: haplotype-aware variant consequences. Bioinformatics.

