# Correction to: Deploying new generation sequencing for the study of flesh color depletion in Atlantic Salmon (*Salmo salar*)

**DOI:** 10.1186/s12864-021-08031-0

**Published:** 2021-09-30

**Authors:** Thu Thi Minh Vo, Tuan Viet Nguyen, Gianluca Amoroso, Tomer Ventura, Abigail Elizur

**Affiliations:** 1 GeneCology Research Centre, University of the Sunshine Coast, Sunshine Coast, Queensland Australia; 2 School of Science, Technology and Engineering, University of the Sunshine Coast, Sunshine Coast, Queensland Australia; 3 School of Biotechnology, International University, Viet Nam National University, Ho Chi Minh, 700000 Vietnam; 4 Centre for AgriBiosciences, AgriBio, Agriculture Victoria, Bundoora, Victoria 3083 Australia; 5 Petuna Aquaculture, East Devonport, Tasmania 7310 Australia

**Correction to:** ***BMC Genomics*** **22, 545 (2021)**

**https://doi.org/10.1186/s12864-021-07884-9**

Following publication of the original article [1], it was reported that an incorrect image was published as Fig.6. The correct Fig. 6 is included in this Correction and the original article has been corrected.

**Fig. 6:**
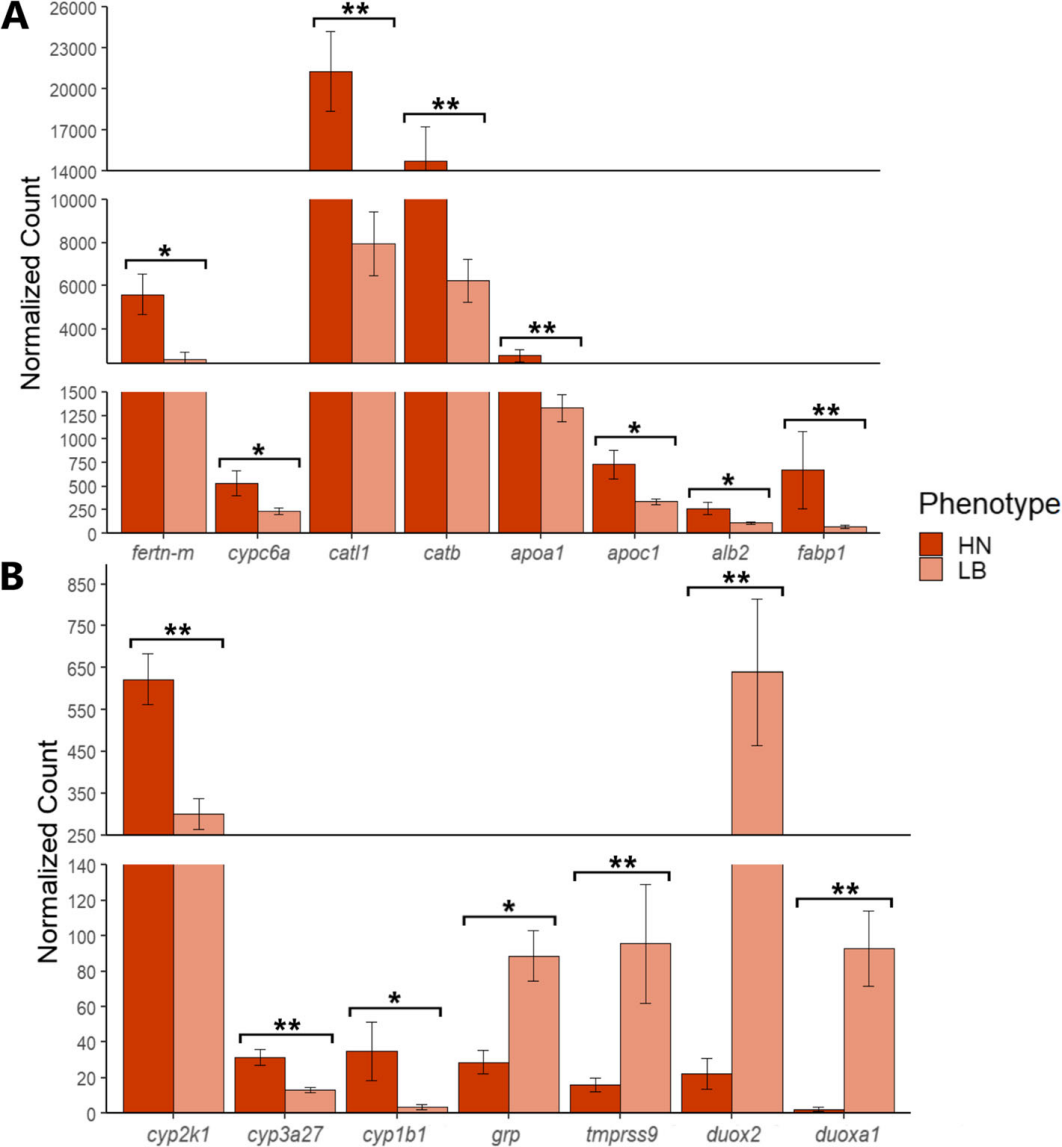
Analysis comparing HN and LB phenotypes. Subset of DEGs of interest identified in the transcriptiomes from the two library preprations. **A** Expression pattern of eight genes identified in the QuantSeq library: ferritin-m (fertn-m), involved in iron ion transport; cytochrome c oxidative subunit 6 A mitochondrial-like (cypc6a), involved in oxidation-reduction process; cathepsin L1 (catl1) and cathepsin B (catb) involved in apoptosis and muscle degradation; apolipoprotein A1 (apoa1), apolipoprotein C1 (apoc1), serum albumin 2 (alb2), fatty acid-binding protein 1 (fabp1) involved in lipid metabolism; **B** Expression pattern of seven genes identified in the TruSeq library: cyp450 gene family cyp2k1, cyp3a27 and cyp1b1, involved in oxidation-reduction process; gastrin-releasing peptide (grp) involved in regulation of feeding, and tmprss9, dual oxidase 2-like (duox2), dual oxidase maturation factor 1-like (duoxa1) involved in SNP analysis. Asterisks (* and **) indicate significant difference between the HN and LB phenotypes at *P* < 0.05 and *P* < 0.01, respectively

## References

[1] Vo TTM, Nguyen TV, Amoroso G, Ventura T, Elizur A (2021). Deploying new generation sequencing for the study of flesh color depletion in Atlantic Salmon (*Salmo salar*). BMC Genomics.

