# Supplementary material for: Deploying new generation sequencing for the study of flesh color depletion in Atlantic Salmon (Salmo salar)
Source: BMC Genomics. 2021 Jul 17;22:545. doi: 10.1186/s12864-021-07884-9 (PMC8285899; doi:10.1186/s12864-021-07884-9)
Supplement: Supplementary file 1 — Additional file 1: Supplementary 1. Distribution of GO terms and the most significant GO terms in comparison of TruSeq and QuantSeq library. [file 12864_2021_7884_MOESM1_ESM.docx]

***Supplementary 1***

**
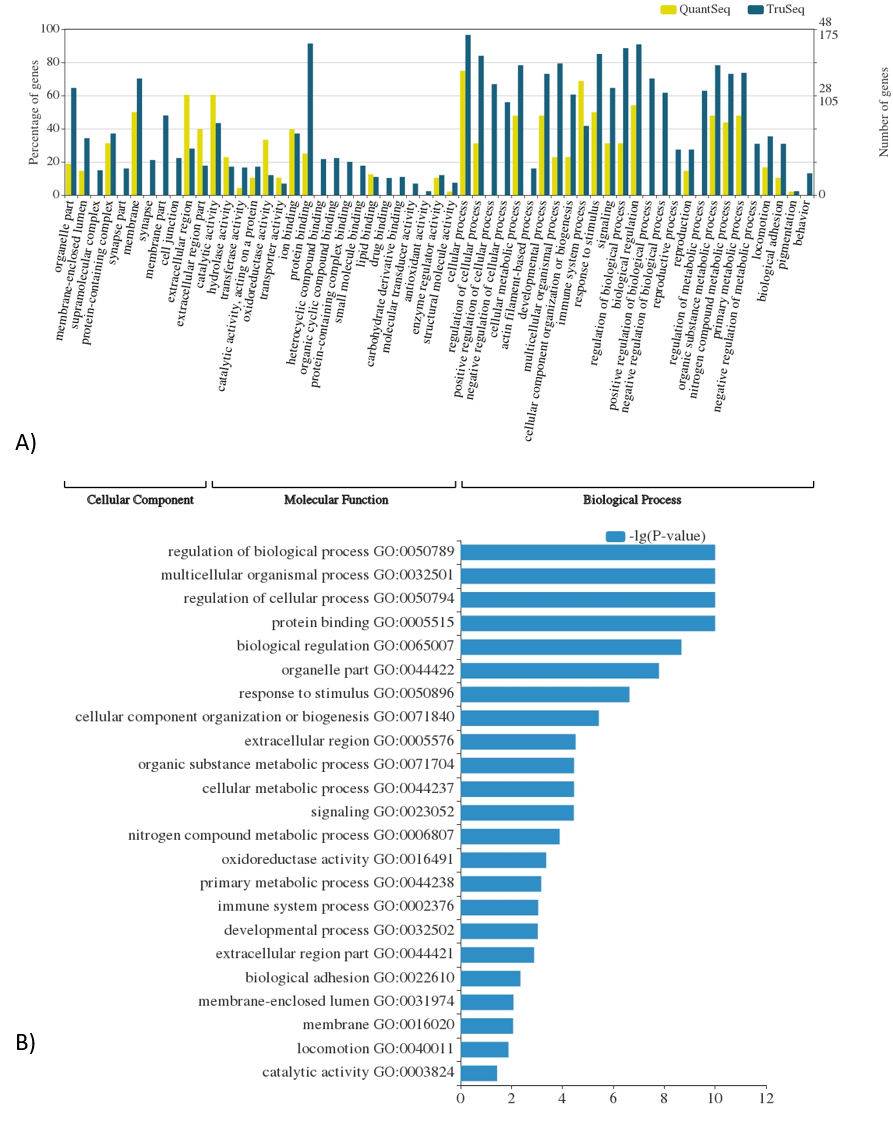
**

**Distribution of GO terms (A) and the most significant GO terms in comparison of TruSeq and QuantSeq library (B).**
