# Supplementary material for: Deploying new generation sequencing for the study of flesh color depletion in Atlantic Salmon (Salmo salar)
Source: BMC Genomics. 2021 Jul 17;22:545. doi: 10.1186/s12864-021-07884-9 (PMC8285899; doi:10.1186/s12864-021-07884-9)
Supplement: Supplementary file 2 — Additional file 2: Supplementary 2. Differentially expressed genes in QuantSeq and TruSeq data. [file 12864_2021_7884_MOESM2_ESM.docx]

***Supplementary 2***

2a. Differentially expressed genes in QuantSeq

| **No.** | **Gene ID** | **Gene name** |
| --- | --- | --- |
| 1 | LOC106590030 | heme oxygenase-like |
| 2 | LOC106610399 | ladderlectin-like |
| 3 | LOC106562274 | cytochrome b-245 light chain-like |
| 4 | LOC106586439 | unconventional myosin-Ib-like, transcript variant X8 |
| 5 | alox5ap | arachidonate 5-lipoxygenase-activating protein, transcript variant X1 |
| 6 | LOC106612300 | ES1 protein homolog, mitochondrial-like |
| 7 | LOC106584721 | sulfide:quinone oxidoreductase, mitochondrial-like |
| 8 | LOC106584698 | dual oxidase 2-like |
| 9 | LOC106602162 | CD209 antigen-like |
| 10 | LOC100136418 | GDP-L-fucose synthase |
| 11 | LOC106569982 | carcinoembryonic antigen-related cell adhesion molecule 6-like |
| 12 | LOC106571947 | glucose-6-phosphate 1-dehydrogenase-like, transcript variant X3 |
| 13 | LOC106563665 | ependymin-like |
| 14 | aqp8 | aquaporin 8 |
| 15 | LOC106612705 | flavin reductase (NADPH)-like |
| 16 | LOC106600949 | ferritin, middle subunit |
| 17 | LOC106600953 | ferritin, middle subunit-like |
| 18 | copt2 | Probable low affinity copper uptake protein 2 |
| 19 | LOC106560595 | transmembrane 4 L6 family member 4-like |
| 20 | LOC106612524 | ribonuclease T2-like |
| 21 | LOC106578314 | angiotensin-converting enzyme-like |
| 22 | nagab | Alpha-N-acetylgalactosaminidase |
| 23 | LOC106568508 | macrophage mannose receptor 1-like |
| 24 | LOC106600955 | ferritin, middle subunit-like |
| 25 | LOC106569806 | solute carrier family 22 member 13-like |
| 26 | npl | N-acetylneuraminate pyruvate lyase (dihydrodipicolinate synthase) |
| 27 | LOC106578169 | fibrinogen alpha chain-like |
| 28 | LOC106578170 | fibrinogen beta chain-like |
| 29 | LOC106590330 | heme oxygenase-like |
| 30 | LOC100136563 | protein AMBP, transcript variant X1 |
| 31 | LOC106600657 | ferritin, middle subunit-like |
| 32 | LOC100136573 | apolipoprotein A-I |
| 33 | LOC106606878 | cytochrome c oxidase subunit 6A, mitochondrial-like |
| 34 | mfi2 | antigen p97 (melanoma associated) identified by monoclonal antibodies 133.2 and 96.5 |
| 35 | LOC106611397 | uncharacterized LOC106611397 |
| 36 | glb1 | galactosidase, beta 1 |
| 37 | LOC106568950 | gamma-interferon-inducible lysosomal thiol reductase-like |
| 38 | fabp1 | fatty acid binding protein 1, liver |
| 39 | LOC106588520 | sialidase-1-like |
| 40 | antithrombin | antithrombin protein |
| 41 | LOC100136922 | serum albumin 2 |
| 42 | mep1b | meprin A, beta |
| 43 | LOC106577511 | apolipoprotein C-I-like |
| 44 | LOC106600764 | ferritin, middle subunit-like |
| 45 | creg1 | cellular repressor of E1A-stimulated genes 1 |
| 46 | LOC106560797 | cathepsin L1-like |
| 47 | ctsb | cathepsin B, transcript variant X1 |
| 48 | LOC101448032 | lysosomal alpha-mannosidase |
| 49 | lgmn | legumain, transcript variant X1 |
| 50 | plac8 | placenta-specific 8 |

2b. Differentially expressed genes in TruSeq

| **No.** | **Gene ID** | **Gene name** |
| --- | --- | --- |
| 1 | LOC106590030 | heme oxygenase-like |
| 2 | LOC106610399 | ladderlectin-like |
| 3 | LOC106562274 | cytochrome b-245 light chain-like |
| 4 | LOC106586439 | unconventional myosin-Ib-like, transcript variant X8 |
| 5 | alox5ap | arachidonate 5-lipoxygenase-activating protein, transcript variant X1 |
| 6 | LOC106612300 | ES1 protein homolog, mitochondrial-like |
| 7 | LOC106584721 | sulfide:quinone oxidoreductase, mitochondrial-like |
| 8 | LOC106584698 | dual oxidase 2-like |
| 9 | LOC106602162 | CD209 antigen-like |
| 10 | LOC100136418 | GDP-L-fucose synthase |
| 11 | LOC106569982 | carcinoembryonic antigen-related cell adhesion molecule 6-like |
| 12 | il18bp | interleukin 18 binding protein |
| 13 | LOC106580289 | alcohol dehydrogenase [NADP(+)] A-like, transcript variant X3 |
| 14 | LOC106583386 | cell division cycle-associated protein 3-like, transcript variant X2 |
| 15 | LOC106589311 | transmembrane protein 106B-like, transcript variant X1 |
| 16 | LOC106560332 | pre-B-cell leukemia transcription factor 1, transcript variant X4 |
| 17 | LOC106566890 | plexin-A1-like, transcript variant X2 |
| 18 | LOC106583903 | uncharacterized LOC106583903, transcript variant X2 |
| 19 | LOC106564918 | uncharacterized LOC106564918, transcript variant X2 |
| 20 | LOC106583774 | growth/differentiation factor 6-A-like |
| 21 | LOC106584836 | immunoglobulin superfamily containing leucine-rich repeat protein 2-like |
| 22 | LOC106584699 | dual oxidase maturation factor 1-like |
| 23 | LOC106564890 | caspase recruitment domain-containing protein 14-like |
| 24 | LOC106604810 | cytochrome b-245 heavy chain-like |
| 25 | LOC106606502 | mitochondrial import inner membrane translocase subunit tim16-like |
| 26 | LOC106575194 | phospholipase A2 inhibitor 31 kDa subunit-like, transcript variant X2 |
| 27 | noxa1 | NADPH oxidase activator 1 |
| 28 | LOC106588435 | caspase recruitment domain-containing protein 14-like |
| 29 | LOC106585586 | indoleamine 2,3-dioxygenase 2-like |
| 30 | LOC106609665 | alpha-N-acetylneuraminide alpha-2,8-sialyltransferase-like |
| 31 | LOC106577496 | actin-binding LIM protein 2-like, transcript variant X9 |
| 32 | LOC106572755 | cytochrome P450 2K1-like, transcript variant X2 |
| 33 | pipox | pipecolic acid oxidase |
| 34 | LOC106564978 | beta-1,3-galactosyl-O-glycosyl-glycoprotein beta-1,6-N-acetylglucosaminyltransferase 3-like |
| 35 | LOC106588966 | serine-aspartate repeat-containing protein F-like, transcript variant X2 |
| 36 | LOC106584748 | protein regulator of cytokinesis 1-like, transcript variant X3 |
| 37 | slc40a1 | solute carrier family 40 (iron-regulated transporter), member 1, transcript variant X1 |
| 38 | LOC106573760 | aminopeptidase N-like |
| 39 | cblb | Cbl proto-oncogene B, E3 ubiquitin protein ligase, transcript variant X1 |
| 40 | LOC106579494 | C-C motif chemokine 20-like, transcript variant X1 |
| 41 | LOC106563668 | putative bifunctional UDP-N-acetylglucosamine transferase and deubiquitinase ALG13, transcript variant X1 |
| 42 | LOC106588461 | microtubule-actin cross-linking factor 1-like, transcript variant X18 |
| 43 | LOC106560550 | complement factor H-like |
| 44 | LOC106578919 | carcinoembryonic antigen-related cell adhesion molecule 1-like, transcript variant X1 |
| 45 | LOC106561458 | programmed cell death 1 ligand 1-like, transcript variant X1 |
| 46 | LOC106604450 | tenomodulin-like |
| 47 | LOC106590295 | probable polyketide synthase 1 |
| 48 | LOC106581253 | SH3 domain-binding protein 5-like |
| 49 | LOC106583798 | 5-aminolevulinate synthase, nonspecific, mitochondrial-like, transcript variant X2 |
| 50 | LOC106562802 | multidrug resistance-associated protein 9-like, transcript variant X1 |
| 51 | LOC106609709 | complement C1q-like protein 2 |
| 52 | LOC106601563 | 3 beta-hydroxysteroid dehydrogenase type 7-like |
| 53 | LOC106578696 | carcinoembryonic antigen-related cell adhesion molecule 20-like |
| 54 | LOC106570315 | adseverin-like, transcript variant X2 |
| 55 | btg3 | B-cell translocation gene 3 |
| 56 | LOC106589020 | ribonuclease ZC3H12A-like |
| 57 | LOC106611598 | ribosome-binding protein 1-like, transcript variant X10 |
| 58 | LOC106602736 | synaptopodin-2-like, transcript variant X2 |
| 59 | LOC106604401 | SH2 domain-containing protein 4A-like, transcript variant X1 |
| 60 | LOC106605117 | ribonuclease ZC3H12A-like, transcript variant X1 |
| 61 | rhbg | Rhesus blood group, B glycoprotein |
| 62 | LOC106571794 | pleckstrin homology domain-containing family G member 1-like, transcript variant X2 |
| 63 | LOC106610701 | kinase D-interacting substrate of 220 kDa-like, transcript variant X2 |
| 64 | LOC106563120 | small nuclear ribonucleoprotein Sm D3 |
| 65 | LOC106563129 | syntaxin-binding protein 1-like, transcript variant X3 |
| 66 | LOC106579928 | apelin receptor A-like |
| 67 | LOC106606647 | NADPH oxidase organizer 1-like |
| 68 | LOC106612689 | CAP-Gly domain-containing linker protein 3-like, transcript variant X2 |
| 69 | LOC106586826 | PTB domain-containing engulfment adapter protein 1-like, transcript variant X3 |
| 70 | LOC106591784 | lysyl oxidase homolog 4-like |
| 71 | LOC106572190 | RING finger protein 223-like |
| 72 | LOC106611235 | complement component C1q receptor-like |
| 73 | LOC106576938 | potassium channel subfamily K member 18-like |
| 74 | LOC106604872 | short transient receptor potential channel 7-like, transcript variant X2 |
| 75 | agtpbp1 | ATP/GTP binding protein 1, transcript variant X9 |
| 76 | LOC106600877 | integrin alpha-IIb-like |
| 77 | LOC106560277 | uncharacterized protein KIAA1211 homolog, transcript variant X3 |
| 78 | LOC106612115 | SH2 domain-containing protein 4A-like, transcript variant X2 |
| 79 | fan1 | FANCD2/FANCI-associated nuclease 1, transcript variant X1 |
| 80 | LOC106561860 | von Willebrand factor-like |
| 81 | LOC106589423 | LIM domain-binding protein 3-like, transcript variant X2 |
| 82 | LOC106588432 | glyceraldehyde-3-phosphate dehydrogenase-like, transcript variant X2 |
| 83 | LOC106589833 | suppressor of cytokine signaling 3-like |
| 84 | birc5 | baculoviral IAP repeat containing 5 |
| 85 | LOC106604703 | sestrin-3-like, transcript variant X3 |
| 86 | LOC106581830 | myosin light chain kinase, smooth muscle-like, transcript variant X5 |
| 87 | LOC106565557 | 6-phosphofructo-2-kinase/fructose-2,6-bisphosphatase 4-like, transcript variant X1 |
| 88 | cep55 | centrosomal protein 55kDa, transcript variant X1 |
| 89 | LOC106589254 | interleukin-22 receptor subunit alpha-2-like, transcript variant X2 |
| 90 | fam198b | family with sequence similarity 198, member B |
| 91 | LOC106575078 | gap junction alpha-5 protein-like, transcript variant X3 |
| 92 | LOC106567285 | protein phosphatase 1 regulatory subunit 12B-like, transcript variant X2 |
| 93 | LOC106602561 | sorbin and SH3 domain-containing protein 2-like |
| 94 | LOC106562345 | leucine-rich repeat-containing protein 49-like, transcript variant X4 |
| 95 | LOC106575652 | carcinoembryonic antigen-related cell adhesion molecule 6-like |
| 96 | LOC106613031 | leucine-rich repeat and fibronectin type III domain-containing protein 1-like, transcript variant X4 |
| 97 | LOC106577687 | TRAF-interacting protein with FHA domain-containing protein A-like |
| 98 | LOC106600618 | BEN domain-containing protein 4-like |
| 99 | LOC106610441 | fatty-acid amide hydrolase 2-A-like |
| 100 | LOC106612644 | putative uncharacterized protein DDB_G0292636, transcript variant X2 |
| 101 | LOC106587226 | uncharacterized LOC106587226, transcript variant X8 |
| 102 | LOC106587071 | kinesin-like protein KIFC3, transcript variant X8 |
| 103 | LOC106572181 | tumor necrosis factor receptor superfamily member 6B-like |
| 104 | LOC106577617 | ladderlectin-like |
| 105 | LOC106566237 | 5-aminolevulinate synthase, nonspecific, mitochondrial-like, transcript variant X2 |
| 106 | LOC106590058 | cadherin-20-like, transcript variant X3 |
| 107 | LOC106564818 | GDP-L-fucose synthase-like |
| 108 | pon2 | Serum paraoxonase/arylesterase 2 |
| 109 | LOC106613900 | tetratricopeptide repeat protein 39A-like, transcript variant X3 |
| 110 | tetn | tetranectin |
| 111 | LOC106567751 | dentin sialophosphoprotein-like |
| 112 | LOC106567787 | rho GTPase-activating protein 20-like, transcript variant X7 |
| 113 | LOC106601165 | myosin-10-like |
| 114 | LOC106606429 | suppressor of cytokine signaling 3-like |
| 115 | LOC106586810 | gremlin-1-like |
| 116 | timp3 | Metalloproteinase inhibitor 3 |
| 117 | fgfp1 | Fibroblast growth factor-binding protein 1 |
| 118 | LOC106566963 | uncharacterized LOC106566963, transcript variant X2 |
| 119 | LOC106575998 | putative interleukin-17 receptor E-like |
| 120 | LOC106599029 | homer protein homolog 3-like, transcript variant X4 |
| 121 | LOC106579692 | probable polyketide synthase 1 |
| 122 | LOC106608979 | E3 ubiquitin-protein ligase TRIM39-like |
| 123 | LOC106599566 | protein LBH-like, transcript variant X1 |
| 124 | LOC106569063 | uncharacterized LOC106569063, transcript variant X2 |
| 125 | LOC106612098 | trichohyalin-like, transcript variant X4 |
| 126 | LOC106604090 | uncharacterized LOC106604090 |
| 127 | LOC106586401 | unconventional myosin-Ib-like |
| 128 | nrp2 | neuropilin 2, transcript variant X1 |
| 129 | LOC106600702 | transmembrane protease serine 9-like |
| 130 | mypn | myopalladin, transcript variant X2 |
| 131 | LOC106566399 | matrix metalloproteinase-9-like |
| 132 | akap6 | A kinase (PRKA) anchor protein 6, transcript variant X3 |
| 133 | b3gnt7 | UDP-GlcNAc:betaGal beta-1,3-N-acetylglucosaminyltransferase 7 |
| 134 | kcnk1 | potassium channel, two pore domain subfamily K, member 1, transcript variant X1 |
| 135 | LOC106567543 | myotonin-protein kinase-like |
| 136 | LOC106599381 | tandem C2 domains nuclear protein-like, transcript variant X2 |
| 137 | LOC106609435 | UPF0577 protein KIAA1324-like |
| 138 | LOC106610245 | ovochymase-2-like, transcript variant X1 |
| 139 | LOC106585050 | claudin-4-like |
| 140 | LOC106567361 | probable glutamate receptor |
| 141 | LOC106607477 | pyruvate dehydrogenase (acetyl-transferring) kinase isozyme 2, mitochondrial-like, transcript variant X2 |
| 142 | LOC106601915 | suppressor of cytokine signaling 3-like |
| 143 | LOC106584658 | uncharacterized protein C11orf96 homolog |
| 144 | LOC106576109 | transmembrane protein 229A-like |
| 145 | zc3h12a | zinc finger CCCH-type containing 12A |
| 146 | LOC106610626 | echinoderm microtubule-associated protein-like 1, transcript variant X5 |
| 147 | LOC106583135 | sortilin-like |
| 148 | grp | gastrin-releasing peptide, transcript variant X2 |
| 149 | LOC106612508 | uncharacterized LOC106612508 |
| 150 | LOC106590316 | uncharacterized LOC106590316 |
| 151 | LOC106589248 | NADPH oxidase organizer 1-like |
| 152 | LOC106581127 | fucolectin-3-like |
| 153 | mob3b | MOB kinase activator 3B, transcript variant X3 |
| 154 | LOC106582808 | ski oncogene-like |
| 155 | LOC106607746 | probable ribonuclease ZC3H12D, transcript variant X2 |
| 156 | LOC106607141 | transcription factor Sox-10-like |
| 157 | LOC106610014 | synaptopodin-2-like, transcript variant X2 |
| 158 | LOC106604568 | alpha-1,3-mannosyl-glycoprotein 4-beta-N-acetylglucosaminyltransferase B |
| 159 | gp182 | G-protein coupled receptor 182 |
| 160 | LOC106583139 | transcription factor EB-like, transcript variant X1 |
| 161 | LOC106560919 | protein NDRG4-like, transcript variant X7 |
| 162 | LOC100136365 | lysozyme C II, transcript variant X1 |
| 163 | LOC106609453 | RAD51-associated protein 1-like |
| 164 | LOC106611485 | arginase-2, mitochondrial-like |
| 165 | LOC106575568 | tensin-like |
| 166 | LOC106578645 | uncharacterized LOC106578645, transcript variant X2 |
| 167 | LOC106609990 | NHP2-like protein 1 |
| 168 | LOC106587409 | lysozyme g-like, transcript variant X1 |
| 169 | LOC106594161 | cytochrome P450 3A27-like |
| 170 | LOC106572101 | laminin subunit beta-2-like, transcript variant X2 |
| 171 | esyt2 | extended synaptotagmin-like protein 2, transcript variant X2 |
| 172 | LOC106564367 | aspartate aminotransferase, cytoplasmic-like |
| 173 | LOC106588137 | multidrug resistance-associated protein 9-like |
| 174 | LOC106580712 | neutrophil cytosol factor 2-like, transcript variant X2 |
| 175 | LOC106564727 | calpain-9-like, transcript variant X1 |
| 176 | LOC106577638 | netrin-3-like |
| 177 | LOC106574088 | leucine-rich repeat-containing protein 15-like |
| 178 | LOC106592009 | Fc receptor-like protein 5, transcript variant X2 |
| 179 | LOC106599706 | cadherin-2-like |
| 180 | LOC106585882 | C-C motif chemokine 4-like |
| 181 | LOC106608810 | interferon-induced very large GTPase 1-like, transcript variant X2 |
| 182 | zdhhc16 | zinc finger, DHHC-type containing 16 |
| 183 | LOC106603794 | myotonin-protein kinase-like, transcript variant X6 |
| 184 | shcbp1 | SHC SH2-domain binding protein 1 |
| 185 | LOC106582397 | GRAM domain-containing protein 1A-like, transcript variant X5 |
| 186 | rnf223 | ring finger protein 223 |
| 187 | LOC106609043 | DEP domain-containing protein 7-like |
| 188 | LOC106607820 | G-protein coupled receptor 126-like, transcript variant X1 |
| 189 | il17d | interleukin 17D, transcript variant X11 |
| 190 | LOC106588497 | mediator of RNA polymerase II transcription subunit 30-like |
| 191 | LOC106610849 | cytochrome P450 1B1-like |
